# Effect of Mechanically Activated Fly Ash, a Superplasticizer, and an Air-Entraining Admixture on the Freeze–Thaw Resistance of Fine-Grained Concrete

**DOI:** 10.3390/ma19173777

**Published:** 2026-09-04

**Authors:** Nazerke Berdikul, Ina Pundienė, Kenzhebek Akmalaiuly, Jolanta Pranckevičienė, Elmira Kurmanbekova, Yerlan Khamza, Aigerim Tolegenova, Assel Kanarbay

**Affiliations:** 1 Department of Construction and Building Materials, Satbayev University, Satbayev Street 22, Almaty 050013, Kazakhstan; k.akmalaiuly@satbayev.university (K.A.); y.khamza@satbayev.university (Y.K.); a.tolegenova@satbayev.university (A.T.); a.kanarbay@satbayev.university (A.K.); 2 Laboratory of Concrete Technology, Institute of Building Materials, Vilnius Gediminas Technical University, Linkmenų Str. 28, LT-08217 Vilnius, Lithuania; ina.pundiene@vilniustech.lt; 3 School of Engineering, International Educational Corporation, Ryskulbekov Street 28, Almaty 050043, Kazakhstan; elmira.kurmanbekowa@yandex.kz

**Keywords:** fly ash, mechanical activation, chemical admixtures, pore structure, freeze–thaw durability, microstructure

## Abstract

This study examines how mechanically activated fly ash (TF), a polycarboxylate superplasticizer (PC), and an air-entraining admixture (AE) affect the hydration, microstructure, pore structure, mechanical properties, and freeze–thaw resistance of fine-grained concrete. Mechanical activation increased the specific surface area of the untreated ash (UF) from 3710 to 6450 cm^2^/g and reduced its mean particle size to 6.06 μm. Replacing 5 wt.% of cement with TF accelerated the nucleation of hydration products and densified the microstructure, as confirmed by exothermic profiling, XRD, and SEM. PC delayed the exothermic peak yet produced the highest heat-release intensity, indicating more efficient wetting and dispersion of reactive surfaces. The TF–PC system reached 39.3 MPa at 28 days, while the ternary TF–PC–AE composition achieved 33.2 MPa with a refined pore-size distribution (mercury intrusion porosimetry). The combined use of PC and AE tripled the pore volume in the 0.01–0.10 μm range and increased it by 28.7% (0.1–1 μm) and 46.21% (1.0–10 μm). The calculated potential freeze–thaw resistance rose from 54.25 to 61.97 and 72.83, confirming the beneficial role of 3–10 μm pores. After 110 accelerated freeze–thaw cycles, the optimized ternary composition lost only 0.55% of its mass.

## 1. Introduction

The durability of concrete structures exposed to freezing and thawing cycles remains a key challenge in construction in cold- and temperate-climate regions. Exposure to freeze–thaw cycles is one of the most aggressive environmental factors, leading to the gradual degradation of cementitious composites and a reduced service life of concrete structures [1,2,3,4]. During the freezing process, the water contained in the pores of the cement stone increases in volume and causes hydraulic and osmotic pressure in the pore space of the material, which leads to the development of internal stresses [5,6]. If the resulting pressure exceeds the tensile strength of the cement matrix, microcracks form and expand during subsequent cycles, causing peeling of the surface, increased porosity, and a gradual decrease in the mechanical strength of the concrete [2,7,8].

Freeze–thaw resistance of concrete, as an important parameter of durability under freeze–thaw conditions, depends on the density and strength of the cement matrix [9,10,11]. Numerous studies indicate a direct correlation between the compressive strength of concrete and its freeze–thaw resistance, as an increase in strength is accompanied by a decrease in permeability and the volume of critically saturated pores [12,13,14,15,16,17,18]. Increased freeze–thaw resistance of fine-grained concrete is achieved by using supplementary cementitious materials (SCMs) and chemical modifiers, primarily air-entraining additives, which create resistance to freeze–thaw cycles [17,19,20]. However, an increase in the content of entrained air is naturally accompanied by a decrease in strength by approximately 4–6% for every 1% of air, which creates a contradiction between the requirements for strength and freeze–thaw resistance [21]. In this regard, the search for solutions that can ensure the required freeze–thaw resistance without significant loss of mechanical characteristics remains a pressing scientific challenge.

In this context, Zeng et al. showed that air-entrained cement paste can be modified with graphene oxide (GO) [22]. Such modification improves freeze–thaw resistance while compensating for strength loss. Nili et al. [23] demonstrated the effectiveness of combining microsilica and air entrainment in improving salt scaling resistance [24,25,26], while the complex influence of the cement matrix structure and active mineral components, or supplementary cementitious materials (SCMs), on the balance between strength and freeze–thaw resistance remains poorly understood.

Along with other SCMs, fly ash is widely used to improve concrete durability. Partial cement replacement (up to 40%) combined with air entrainment has been shown to improve resistance to freeze–thaw cycles and sulfate attack, with the optimal effect achieved with 5% to 20% high-calcium ash [27,28,29,30]. For de-icing-salt exposure in particular, a properly designed air-void system allows fly-ash concrete to meet the accepted surface-scaling limits, although the fly ash can hinder air entrainment and requires a carefully controlled admixture dosage [31]. Mechanical activation of fly ash intensifies the pozzolanic reaction, accelerates the binding of calcium hydroxide, and promotes the formation of a denser cement matrix, which has a positive effect on strength characteristics [32,33,34,35]. However, despite the established strength-enhancing benefits of fly ash activation, the combined effect of fly ash activation with air-entraining admixtures on the freeze–thaw resistance of concrete remains poorly understood, indicating a scientific gap and the need for further research.

As shown in many studies [36,37], partial replacement of Portland cement with fly ash improves the workability and rheological characteristics of mixtures, reduces water separation and heat generation, and, due to the pozzolanic interaction of the amorphous phase of ash with Ca(OH)_2_, promotes the formation of additional C–S–H gel and compaction of the microstructure of concrete [38,39]. However, replacing cement with mineral components alters the water requirement and particle size distribution of the system, necessitating additional optimization of the mixture’s rheological parameters. Thus, increasing the dispersion of the system upon introducing mineral additives requires targeted regulation of interparticle interactions and the distribution of water throughout the mixture. Polycarboxylate-based superplasticizers effectively address this issue. Their adsorption-steric mechanism of action ensures stable dispersion of cement particles, reduces flocculation, and reduces the water-cement ratio without compromising workability [40,41,42,43,44]. It has been shown that the use of PC substantially reduces water consumption and increases the density of cement paste, thereby increasing the strength and durability of concrete [45,46,47].

Despite a significant body of research on the individual effects of fly ash [27,28,29,30], polycarboxylate-based superplasticizers [40,41,42,43,44,45], and air-entraining admixtures [17,19,20], their combined application in multicomponent systems incorporating mechanically activated fly ash has not been systematically investigated, indicating a clear research gap.

There is limited understanding of the extent to which cement matrix compaction due to fly ash activation and the reduction in water-cement ratio when using superplasticizers can compensate for the reduction in strength caused by air entrainment while simultaneously improving freeze–thaw resistance. To date, there are no systematic studies that comprehensively assess the performance of such systems.

This study aims to evaluate the combined effect of mechanically activated fly ash, superplasticizers, and an air-entraining admixture on the properties of fine-grained concrete. Particular attention is paid to the influence of these components on hydration processes, water absorption, strength, and freeze–thaw resistance.

The working hypothesis is that the use of mechanically activated fly ash in combination with superplasticizer allows an increase in the density of the cement matrix, which partially offsets the decrease in strength due to the introduction of an air-entraining admixture, while also improving the freeze–thaw resistance of the concrete.

The scientific novelty of this study lies in the comprehensive assessment of the effects of mechanically activated fly ash and chemical admixtures on the performance properties of fine-grained concrete, as well as in the establishment of a rational combination of components that balances strength and freeze–thaw resistance. The obtained results can be used to develop durable, fine-grained concrete compositions using industrial waste and modern chemical additives for harsh-climate applications.

## 2. Materials and Methods

### 2.1. Materials

In this study, Portland cement CEM I 42.5 N (Bukhtarma Cement Company LLP, HeidelbergCement Group, Ust-Kamenogorsk, Kazakhstan), conforming to EN 197-1:2011 [48], was used as the primary binder. The mineral composition of the cement was dominated by tricalcium silicate (C_3_S—58.54%), along with dicalcium silicate (C_2_S—15.29%), tricalcium aluminate (C_3_A—10.4%), and tetracalcium aluminoferrite (C_4_AF—10.17%). The chemical composition included CaO (63.5%), SiO_2_ (20.76%), Al_2_O_3_ (6.12%), and Fe_2_O_3_ (3.37%), with a total alkali content (Na_2_O + K_2_O) of 1.03% and loss on ignition of 0.30%.

**Figure 1:**
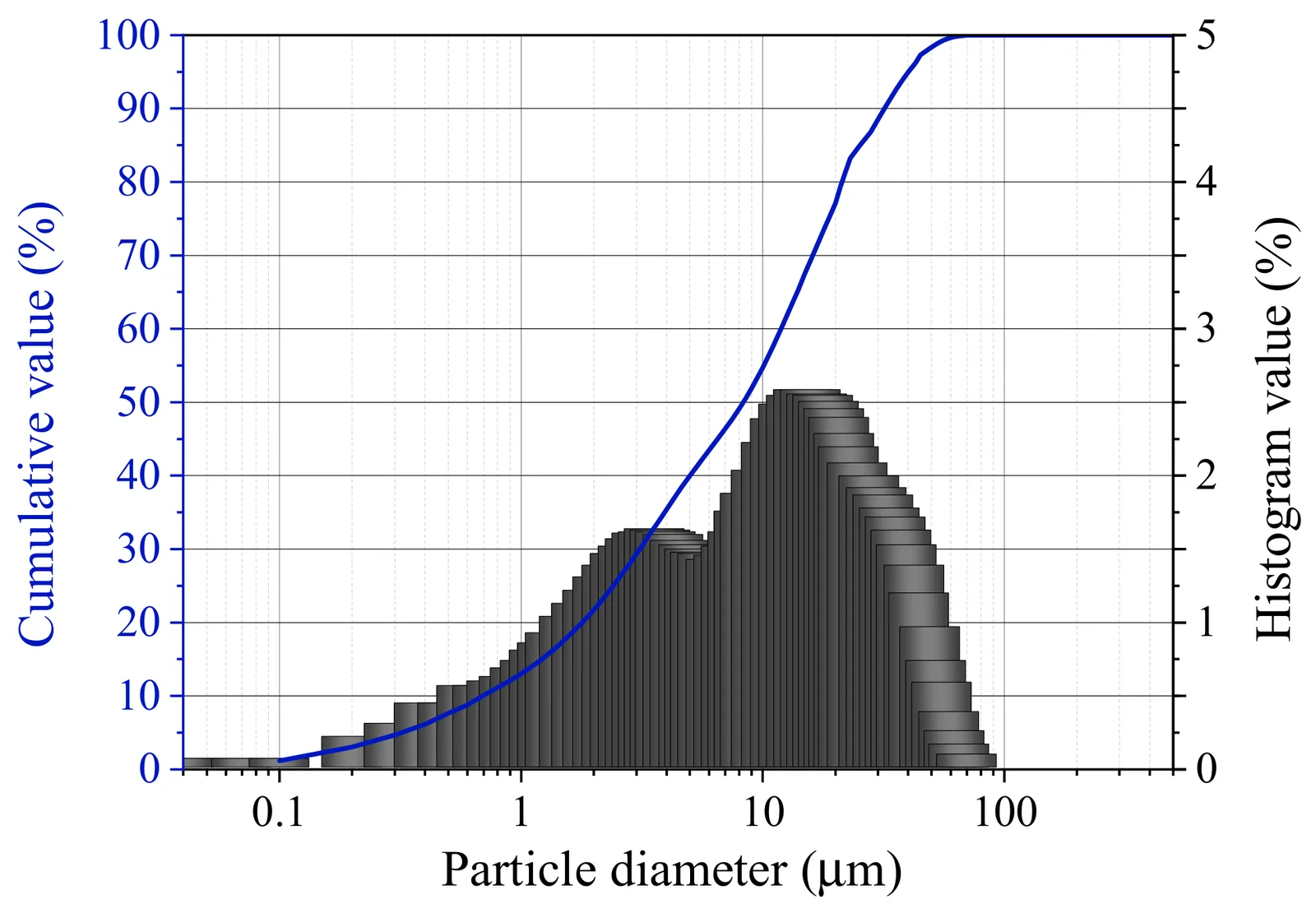
Particle size distribution of cement.

The specific surface area of the cement was 4010 cm^2^/g (Blaine), with a bulk density of 1.1 g/cm^3^. The initial and final setting times were 140 and 190 min, respectively. The particle size ranged from 0.1 to 70 μm, with characteristic values of d_10_ = 0.69 μm, d_50_ = 8.31 μm, and d_90_ = 31.89 μm. The particle size distribution is presented in Figure 1.

Coal fly ash obtained from a coal-fired thermal power plant was used as a supplementary cementitious material in both untreated (UF) and mechanically activated (TF) forms. The untreated fly ash was predominantly composed of aluminosilicate phases. Based on the ratio of basic oxides (Fe, Ca, Mg, Na, and K) to acidic oxides (Si, Al, and Ti), equal to 0.033, the ash was classified as acidic. However, due to the amount of SiO_2_, Al_2_O_3_, and Fe_2_O_3_ exceeding 70%, it can also be categorized as fly ash (Class F) [49].

**Figure 2:**
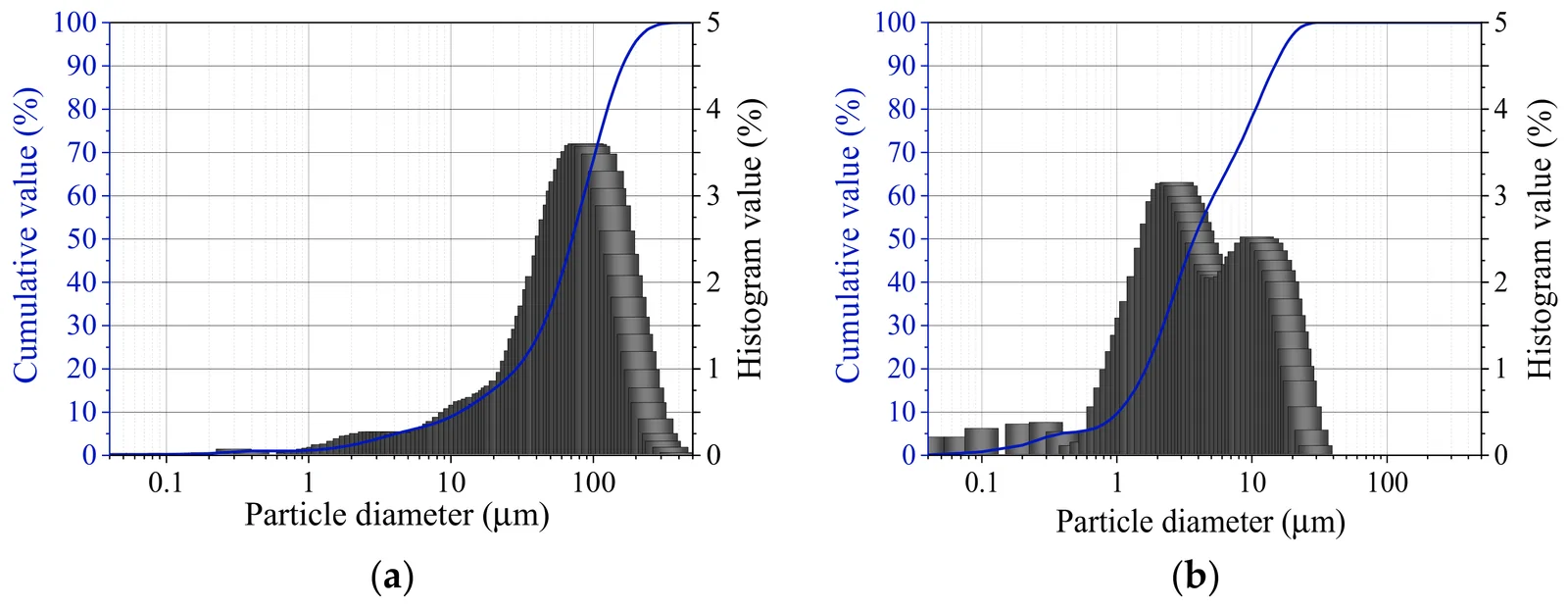
Particle size distribution of fly ash: (**a**) untreated (UF); (**b**) mechanically activated (TF).

**Figure 3:**
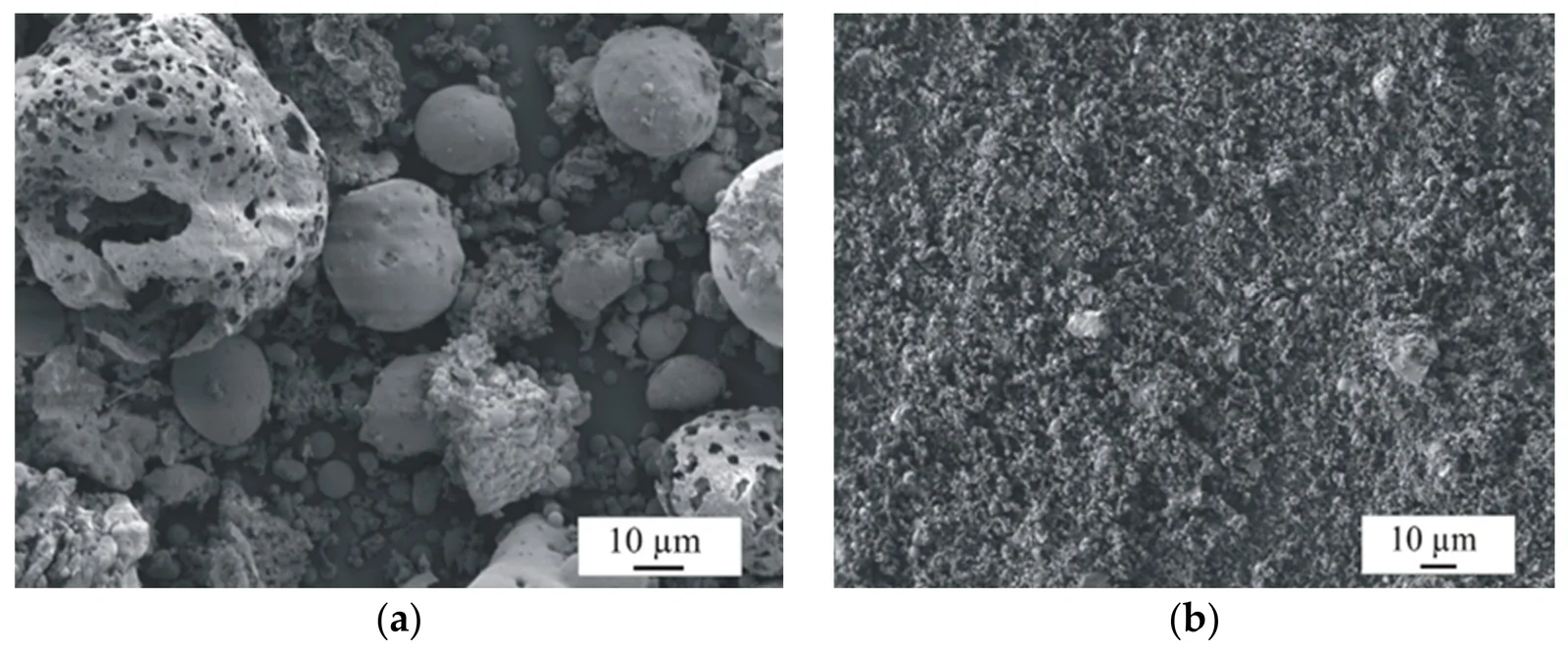
SEM images of fly ash: (**a**) untreated (UF); (**b**) mechanically activated (TF).

The specific surface area of UF was 3710 cm^2^/g, with a true density of 2.2 g/cm^3^ and a bulk density of 0.794 g/cm^3^. The loss on ignition at 1000 °C was 5.2%. The particle size distribution showed that d_10_ = 11.53 μm, d_50_ = 71.11 μm, and d_90_ = 160.82 μm, with an average particle size of 80.93 μm (Figure 2a). SEM analysis (Figure 3a) revealed that the particles were mainly spherical with either smooth or porous surfaces, typical of high-temperature combustion products [50].

**Figure 4:**
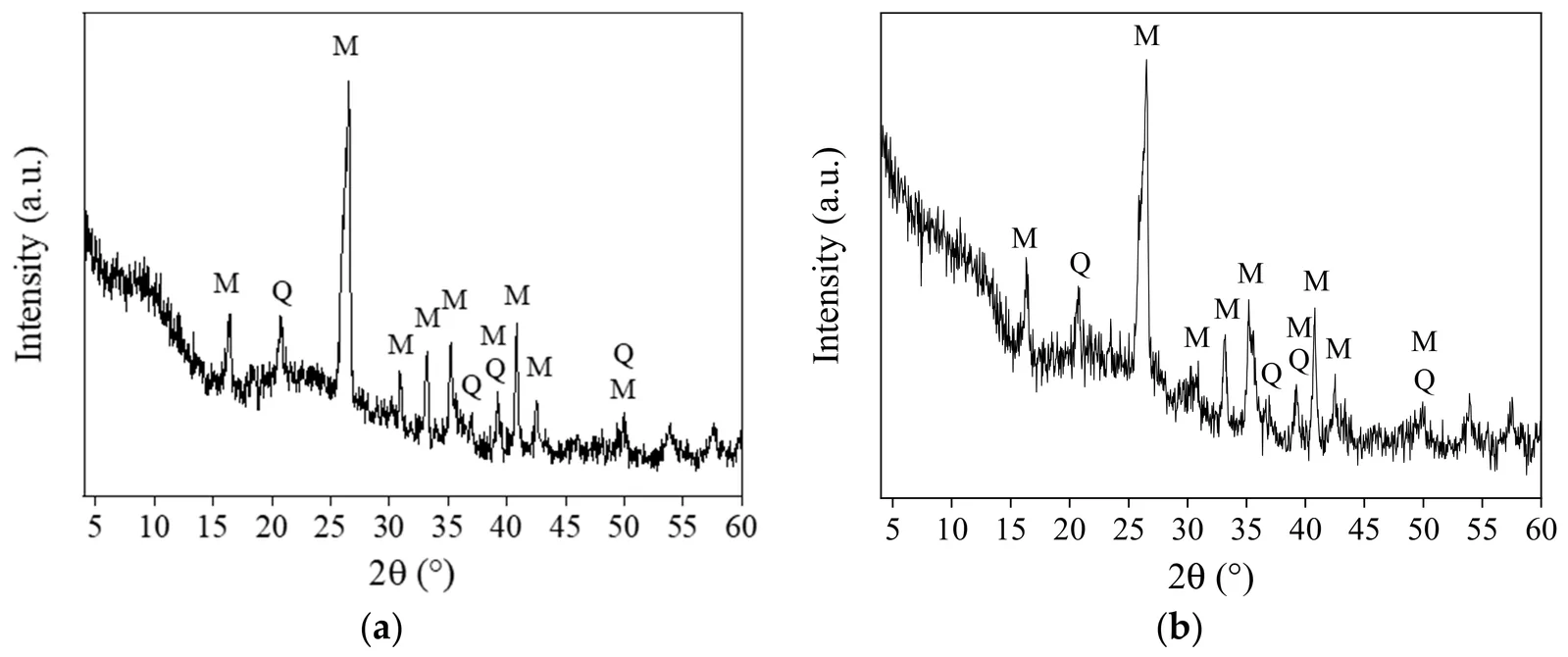
X-ray diffraction patterns of fly ash: (**a**) untreated (UF); (**b**) mechanically activated (TF). Q—Quartz; M—Mullite.

X-ray diffraction analysis (Figure 4a) indicated that the main crystalline phases were quartz (SiO_2_) and mullite (3Al_2_O_3_·2SiO_2_ A broad hump centred at approximately 15–30° 2θ confirmed the presence of a pronounced amorphous phase; consistent with the XRF composition, in which SiO_2_ and Al_2_O_3_ together account for about 89 wt.%, and with the predominance of mullite, this halo corresponds to an aluminosilicate glass typical of Class F fly ash rather than to amorphous silica alone [51].

Mechanical activation of fly ash was carried out in a planetary ball mill for 7 h at a specified ball-to-powder ratio. As a result, the specific surface area increased to 6450 cm^2^/g, accompanied by changes in density. The particle size distribution was markedly refined, with no particles exceeding 30.73 μm. The characteristic values were d_10_ = 0.92 μm, d_50_ = 3.72 μm, and d_90_ = 14.73 μm, with an average particle size of 6.06 μm (Figure 4b).

SEM images (Figure 3) showed that mechanical activation resulted in the breakdown of spherical particles into angular fragments measuring approximately 3–4 μm. XRD analysis of TF (Figure 4b) confirmed the same primary phases as UF, but with reduced crystallinity and peak broadening due to structural disorder induced by intensive grinding. This process increased the amorphous phase content and is expected to enhance the material’s pozzolanic reactivity.

The chemical compositions of untreated fly ash (UF) and mechanically activated fly ash (TF) are presented in Table 1. Both materials are predominantly composed of SiO_2_ and Al_2_O_3_, confirming their aluminosilicate nature. After mechanical activation, a slight increase in the content of SiO_2_ and Al_2_O_3_ is observed, which can be attributed to particle refinement, sample inhomogeneity, and LOI difference.

The CaO content remains relatively low in both cases. At the same time, the loss on ignition (LOI) decreases from 5.2% for UF to 4.2% for TF, indicating partial removal of volatile components and improved reactivity of the material. The increase in SiO_2_ and Al_2_O_3_ content, combined with the enhanced specific surface area, is expected to contribute to the higher pozzolanic activity of the mechanically activated fly ash.

### 2.2. Chemical Admixtures

A polycarboxylate-based superplasticizer, Sika^®^ 17 (PC) (Sika AG, Baar, Switzerland), in liquid form and brown in color, was used to reduce water demand and enhance particle dispersion within the cementitious system. The admixture had a density of 1.075–1.115 g/cm^3^ at 20 °C and a pH of 4.0–7.0, according to the technical specifications.

UFFAPORE TCO, an air-entraining admixture (AE), is a white powder based on sodium alkenesulfonate. Upon addition to the cement mix, it promotes the formation of a stable and uniformly distributed air-void system within the cementitious matrix. The dosages of all chemical admixtures were defined as a percentage of the cement mass.

### 2.3. Aggregates and Water

**Figure 5:**
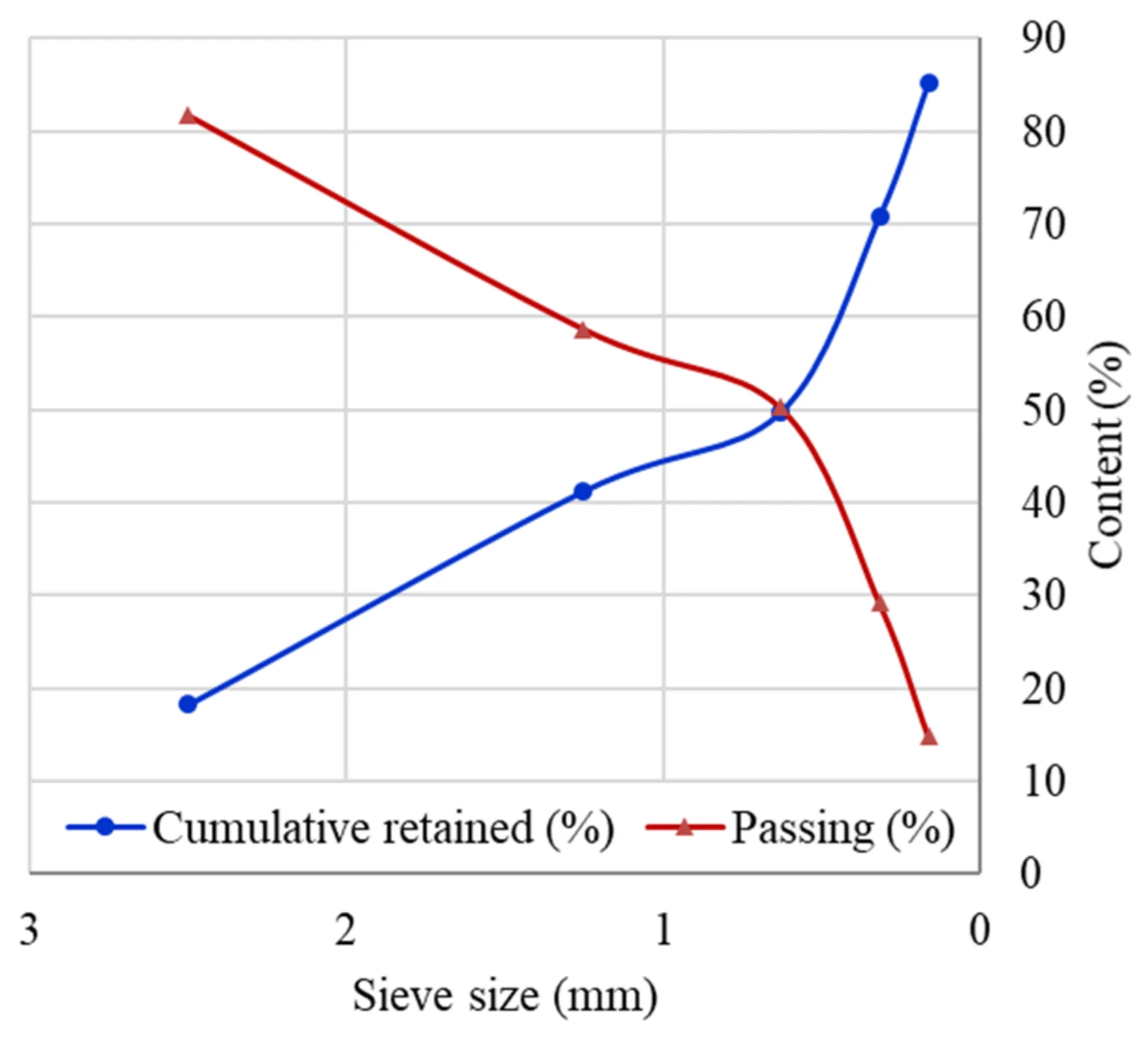
Grain-size distribution of the quartz sand used as fine aggregate.

Natural quartz sand sourced from a local quarry was used as fine aggregate. The principal physical and mechanical properties of the sand are summarized in Table 2. The sand exhibited a fineness modulus of 2.66, a particle density of 2620 kg/m^3^, and a bulk density of 1630 kg/m^3^. The clay and dust content did not exceed 0.9%, confirming the aggregate’s suitability for concrete production. The void ratio was 38.2%, and the water demand was 5.4%, both of which are considered in the assessment of mixture workability and water requirement. The grain-size distribution of the sand is shown in Figure 5, and its fineness modulus of 2.66 confirms a medium sand suitable for fine-grained concrete. The particle-size characteristics of the sand ensured adequate packing density in the fine-grained concrete system. Potable water complying with the requirements for concrete production was used in all mixtures.

### 2.4. Experimental Program

The experimental program was structured into two consecutive stages to assess the effects of mineral and chemical modifiers at different levels of material complexity, from the binder matrix to fine-grained concrete.

The first stage was carried out on cement paste systems without sand. This approach enabled us to minimize the influence of fine aggregate and assess more directly the contributions of UF, TF, and chemical admixtures to early hydration, heat evolution, and initial structure formation in the cement stone. The main objective of this stage was to determine the influence of UF and TF on the exothermic profile during hydration in the investigated compositions, on mineral formation during the hydration process, and to select the most effective formulations for further development.

In the second stage, the most promising compositions identified at the cement paste level were used to design fine-grained concrete mixtures with sand as the fine aggregate. This transition from a simplified binder system to a multicomponent concrete matrix enabled evaluation of the selected modifiers under conditions closer to practical use. Particular emphasis was placed on the rheological properties of fresh mixtures, as well as on the physical-mechanical and durability-related characteristics of the hardened material. This two-stage design first isolates the intrinsic contribution of each modifier to hydration and structure formation in the sand-free pastes, unobscured by the aggregate, and then advances only the most effective compositions to evaluation as fine-grained concrete under conditions approaching practical use.

Overall, the adopted research strategy provided a rational and scientifically supported framework for composition optimization, linking the early hydration behavior of cement paste systems with the subsequent performance of fine-grained concrete.

### 2.5. Cement Paste Systems: Preparation and Test Methods

For the exothermic profile and SEM and XRD studies, simplified sand-free cementitious systems were designed to isolate the effects of mineral and chemical modifiers on the early hydration behavior of the binder matrix. The experimental program included one reference Portland cement paste and eighteen modified compositions containing untreated fly ash (UF), mechanically activated fly ash (TF), a polycarboxylate ether-based superplasticizer (PC), an air-entraining admixture (AE), and selected combinations thereof (Table 3). The replacement levels of UF and TF ranged from 5 to 15 wt.% of cement, while PC was introduced at 0.5, 1.0, and 1.5 wt.%, and AE at 0.15, 0.30, and 0.45 wt.%.

The selected UF and TF contents were based on literature reports and the author’s previous findings [52], which indicate that excessive cement replacement may markedly retard hydration. Accordingly, this stage of the study was intended to assess the efficiency of fly ash mechanical activation and to determine the influence of the selected modifier systems on paste spreadability, setting time, exothermic profile (EXO), and XRD characteristics of the cement paste. To this end, paste spreadability, setting time, and the EXO were evaluated, with the latter serving as an informative indicator of early hydration kinetics and initial structure formation.

#### 2.5.1. Paste Preparation and Mix Design

For comparative analysis, the mixtures were tested with a fixed water-to-cement ratio of 0.27. All raw materials were weighed and mixed under controlled laboratory conditions according to a standardized procedure.

#### 2.5.2. Flowability and Setting Time

**Figure 6:**
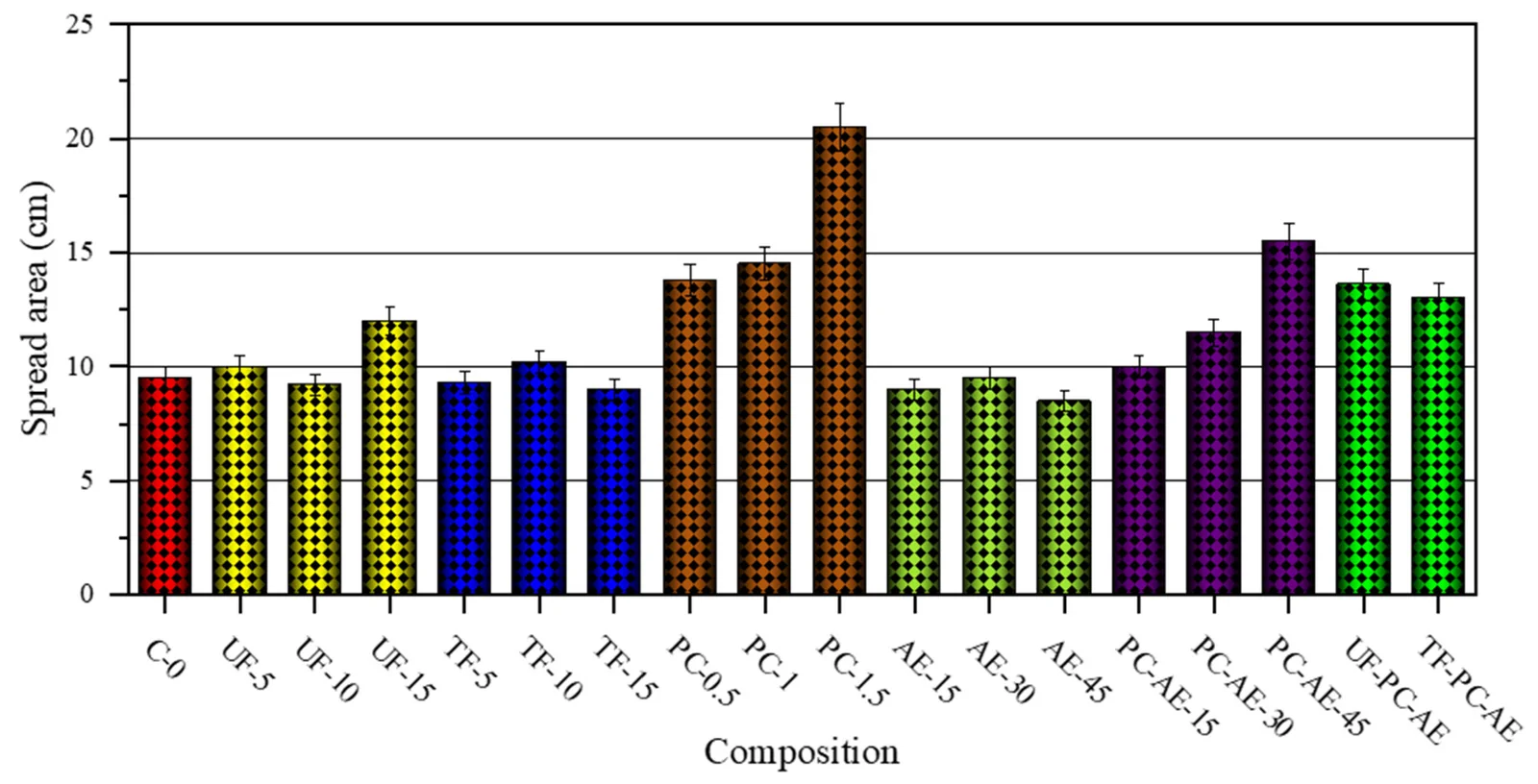
Effect of mineral and chemical admixtures on the spread diameter of fresh cement paste. Error bars represent ± one standard deviation (n = 3).

**Figure 7:**
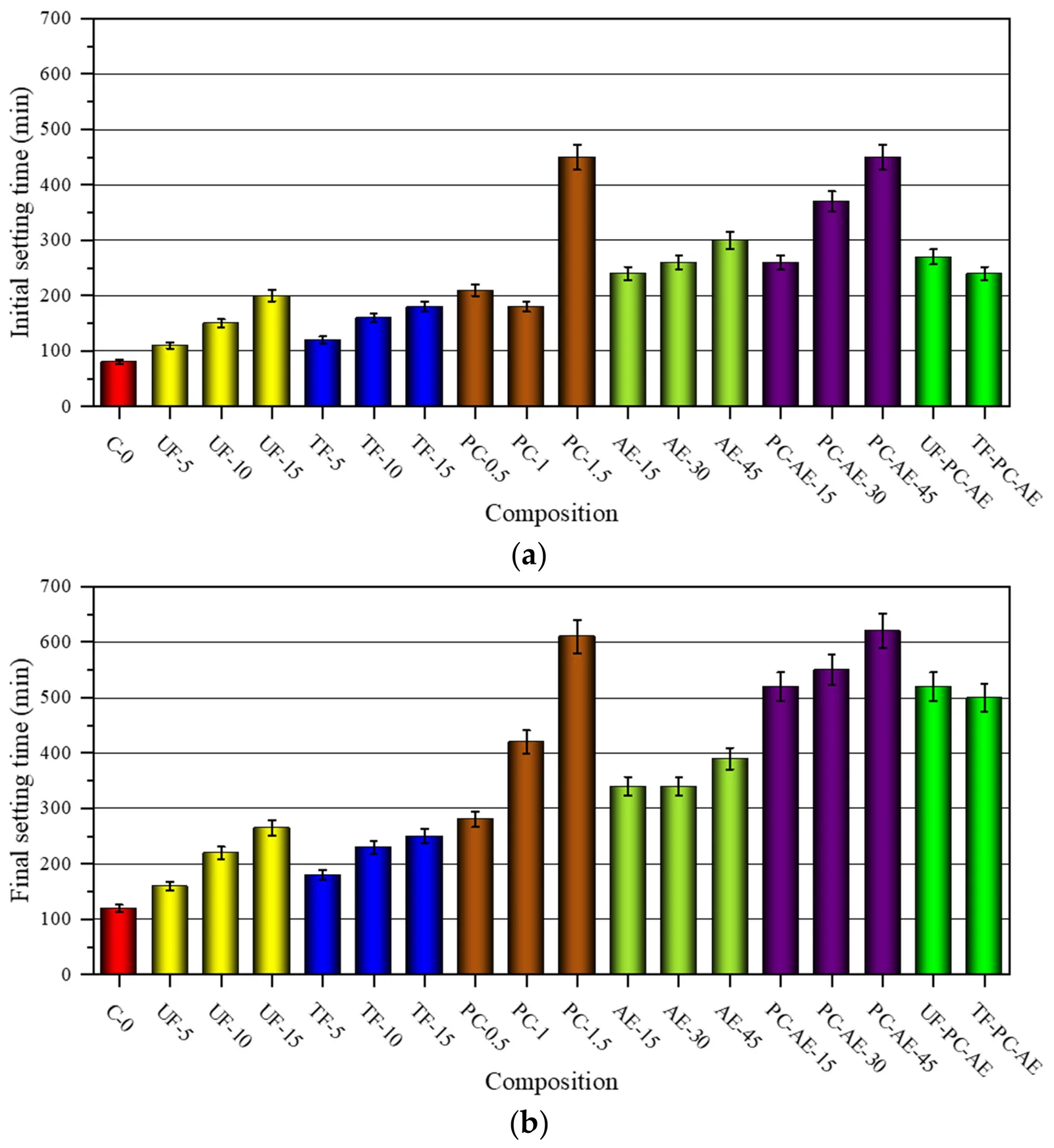
Initial (**a**) and final (**b**) setting times of cement pastes containing UF, TF, and different chemical admixtures. Error bars represent ± one standard deviation (n = 3).

Paste flowability was assessed using a mini-slump test with a Vicat cone on a glass plate. The cone had a height of 40 mm, an upper diameter of 70 mm, and a lower diameter of 80 mm. Immediately after lifting the cone, the paste was allowed to spread freely, and the spread diameter was measured. Setting behavior was determined by the Vicat method in accordance with EN 196-3 and ASTM C191 [53,54]. The initial and final setting times were recorded to evaluate the influence of mineral and chemical modifiers on the early hardening characteristics of the cement paste systems. For each mix, the spread and the setting times were determined on three independent specimens; the values reported in Figure 6 and Figure 7 are the mean values (n = 3), and the error bars represent ± one standard deviation.

#### 2.5.3. Exothermic (Hydration Heat) Measurements

The exothermic behavior of the investigated pastes was assessed by recording temperature evolution during the early hardening stage, in accordance with the ALCOA methodology [55], and was evaluated according to [56]. The heat generated during the exothermic reactions of cement minerals was measured at 20 °C using 1.5 kg specimens placed in an insulated 10 × 10 × 10 cm textolite mould. A type T thermocouple was embedded in the specimen and connected to a data-acquisition system to record temperature over time continuously.

#### 2.5.4. Microstructure and Phase Analysis (SEM and XRD)

The morphology of the cement paste systems was examined using a JEOL JSM-7600F scanning electron microscope (SEM) (JEOL Ltd., Tokyo, Japan) with a resolution of 2.0 nm. The analyzed compositions included the reference paste, pastes incorporating untreated fly ash (UF) or activated fly ash (TF) at replacement levels of 5, 10, and 15%, as well as mixtures modified with PC, AE, and their combination. The chemical admixtures were introduced at different dosages to evaluate their influence on the formation and morphology of hydration products and on the overall microstructural development of the paste systems.

### 2.6. Design and Preparation of Fine-Grained Concrete Mixtures

Following the binder-stage investigation, seven fine-grained concrete mixtures were designed to verify the effect of mineral and chemical modification in a multicomponent system more representative of practical application. The experimental matrix consisted of one control mixture (K) and six modified compositions containing fly ash, a PC, and AE, and selected combinations thereof (Table 4). Portland cement served as the main binder, quartz sand as the fine aggregate, and fly ash as a partial cement substitute.

The reference composition consisted of 450 g of cement, 1550 g of sand, and 230 g of water. In the modified systems, fly ash was incorporated at 5 wt.% of cement, PC at 1 wt.%, and AE at dosages of 0.15 and 0.30 wt.%. In addition to mixtures containing individual modifiers, combined systems comprising fly ash with PC and fly ash with PC plus AE were also prepared to examine potential synergistic interactions between mineral and chemical modification.

All raw materials were weighed and mixed under controlled laboratory conditions using a standardized preparation procedure. The sequence of component addition and all processing parameters were maintained constant for all mixtures to ensure the reproducibility and comparability of the experimental results. This experimental design provided a consistent basis for assessing the influence of fly ash and admixture combinations on the fresh-state characteristics and hardened properties of fine-grained concrete.

### 2.7. Test Methods

#### 2.7.1. Compressive Strength

The physical-mechanical characteristics of the fine-grained concrete were determined after 7 and 28 days of curing. Among these, compressive strength was assessed on 100 × 100 × 100 mm concrete cubes after water curing in accordance with EN 12390-3 [57]. Specimen preparation and curing were performed in accordance with EN 12390-2 [58], while the tests were conducted using an automatic Pilot 4.2000 (Controls S.p.A., Milan, Italy) testing machine equipped with a dual-piston loading system.

#### 2.7.2. Water Absorption

Water absorption kinetics were assessed by continuous hydrostatic weighing or stepwise gravimetric measurement of pre-dried concrete specimens over 24 h in accordance with BS 1881-122:2011 [59]. Before testing, all specimens were dried to constant mass, and their initial dry mass, *m_c_* (g), was recorded. The mass increase associated with water uptake was then measured at specified time intervals throughout the test. After 24 h of exposure, the mass of the saturated specimen, *m*_24_ (g), was determined.

The 24 h water absorption was calculated according to Equation (1):

(1) $$W_{24}=\frac{m_{24}-m_{c}}{m_{c}}\times 100,\%.$$

where *m_c_* is the dry mass of the specimen, g, and *m*_24_ is the mass of the specimen after 24 h of water saturation, g.

In the continuous hydrostatic weighing mode, the time-dependent mass increase was used to construct the water absorption kinetics curve. Upon completion of the test, the saturated specimens were weighed both in air and hydrostatically, and their volume was determined in accordance with EN 12390-7:2019 [60].

#### 2.7.3. Porosity Determination

Mercury intrusion porosimetry (MIP) measurements were performed using a Pore Master PM33-12 porosimeter (Quantachrome Instruments, Boynton Beach, FL, USA). Specimens for analysis were extracted from the internal layer of the hardened concrete samples to ensure representative characterization of the bulk pore structure. For this study, samples were taken from the concrete matrix to prevent sand particles from entering the sample. For this purpose, the matrix part (cement, TF, and additives, W/DC (water-to-dry components) 0.27) was prepared and kept for 28 days under the same conditions as the concrete samples.

The prediction of freeze–thaw resistance in concrete based on pore-structure parameters has been validated in several studies [61,62]. In the present work, the indirect method was employed to evaluate the potential freeze–thaw resistance of the investigated fine-grained concrete compositions.

Potential freeze–thaw resistance number (Fc)—method is based on the approach developed by Maage [62], in which the potential freeze–thaw resistance is evaluated using a single numerical index calculated from MIP data according to Equation (2):

Fc = (3.2/PV) + (2.4 × P3)(2)

where Fc is the freeze–thaw resistance number (dimensionless); PV is the total pore volume (cm^3^/g);

P3 is the percentage of pores with diameters larger than 3 μm (%).

Pores with diameters exceeding 3 μm are generally not readily filled with water under ambient conditions and therefore act as buffer reservoirs that accommodate the volumetric expansion of freezing water, thereby reducing internal stress. Accordingly, a higher Fc value indicates a more favorable pore geometry with respect to freeze–thaw resistance.

#### 2.7.4. Freeze–Thaw Resistance Testing

Freeze–thaw resistance was evaluated using an accelerated freeze–thaw procedure based on the cube test approach adapted from CEN/TS 12390-9 [63], with exposure in a 5% NaCl solution. The use of a 5% NaCl solution provided more severe exposure conditions than freezing and thawing in water. It enabled evaluation of the mixtures’ durability under saline freeze–thaw action, which is relevant for concrete exposed to deicing salts in cold regions. A salt solution rather than pure water was used because chloride raises the osmotic pressure and the degree of critical saturation of the pores and lowers and broadens the freezing point, which promotes layer-by-layer freezing and surface scaling and thus reproduces the most aggressive de-icing-salt service condition. Therefore, the procedure was used to assess the relative resistance of the investigated fine-grained concrete mixtures to cyclic freezing and thawing under severe saline conditions. For each of the seven mixtures, six concrete cubes measuring 100 × 100 × 100 mm were prepared and tested after 28 days of curing. Before cyclic exposure, the specimens were saturated in a 5% NaCl solution. The test involved repeated freezing of the specimens in air at −18 ± 2 °C, followed by thawing in a 5% NaCl solution at 20 ± 2 °C. The onset of freezing was defined as the point at which the chamber temperature reached −16 °C. The 5% NaCl solution was not replaced during the test; the solution level was checked at each inspection to keep the immersion depth constant. In the present study, the performance of the mixtures was assessed after 8, 13, 20, 30, 45, 75, and 110 accelerated freeze–thaw cycles. The intermediate testing points after 8 and 13 cycles were introduced to monitor the early evolution of mass loss and compressive strength during cyclic exposure. At each testing stage, the specimens were removed from the chamber, inspected for visible surface deterioration, wiped to remove surface moisture, weighed, and subsequently tested in compression. The mass-loss and compressive-strength results are reported as mean values, with error bars representing ± one standard deviation. The durability performance was evaluated based on three criteria: mass loss, compressive strength retention, and visual surface condition. The results were interpreted by comparing the changes in these parameters after cyclic exposure rather than by assigning a freeze–thaw resistance grade. Since no frost-resistance grade was assigned in this study, the highest applied exposure level was reported as 110 accelerated freeze–thaw cycles in a 5% NaCl solution. Mixtures were considered to retain satisfactory freeze–thaw performance when mass loss remained limited, compressive strength was maintained at a satisfactory level, and no critical visual damage, such as cracking, spalling, or severe surface scaling, was observed.

## 3. Results and Discussion

### 3.1. Fresh-State Properties, Setting Behavior, and Early Hydration of Cement-Based Systems

The fresh-state properties and setting behavior of the investigated cement-based compositions were analyzed to clarify the influence of untreated fly ash (UF), activated fly ash (TF), polycarboxylate ether superplasticizer (PC), and air-entraining admixture (AE) on the early-stage performance of the studied mixture. Particular attention was paid to mixture spread, initial and final setting times, as these parameters largely determine the system’s technological behavior, the onset of structure formation, and the subsequent development of the physical and mechanical properties of hardened concrete.

As shown in Figure 6, the spread values varied considerably depending on the type and dosage of the modifying components. The reference composition C-0 exhibited a spread of 9.7 cm. The incorporation of UF did not produce a monotonic trend: the spread values were 9.9, 9.2, and 12.1 cm for UF-5, UF-10, and UF-15, respectively. This behavior suggests that the combined influence of dilution, particle packing, and water demand governed the effect of UF on workability. In contrast, the TF-modified pastes showed more restrained flowability, with spread values of 9.4, 10.2, and 9.1 cm for TF-5, TF-10, and TF-15, respectively. In general, TF did not improve mixture mobility and, at the higher replacement level, tended to reduce it, which may be attributed to its higher fineness and larger specific surface area. The most pronounced effect on the spread was observed in the PC-containing pastes. As the PC dosage increased from 0.5 to 1.5%, the spread increased from 13.5 to 14.6 cm and 20.9 cm, respectively, demonstrating a clear dosage-dependent plasticizing effect. This response is consistent with PC’s ability to deflocculate cement particles and release water trapped within the coagulated structure of the paste.

In contrast, the AE-modified mixtures remained close to the control level, with spread values of 8.9, 9.6, and 8.4 cm for AE-15, AE-30, and AE-45, respectively, indicating that the air-entraining admixture had only a limited influence on fresh-state consistency.

For the combined PC-AE systems, the spread values were 10.0, 11.2, and 15.6 cm for PC-AE-15, PC-AE-30, and PC-AE-45, respectively, indicating a non-linear interaction between the plasticizing and air-entraining components. At the lowest AE dosage, the spread decreased markedly relative to the PC-only paste, and it increased progressively with further increases in AE content.

Having established the effect of the investigated components on paste consistency, the subsequent analysis focused on their influence on the setting characteristics and hydration kinetics of the modified cement pastes. The setting time results (Figure 7) clearly demonstrate that all investigated admixtures influenced both the initial and final setting of the cement paste relative to the control mixture. In the fly ash-containing pastes, both UF and TF led to a progressive increase in setting time with increasing replacement level. For the UF series, the initial setting time increased from 81 min for the control to 110, 150, and 200 min for UF-5, UF-10, and UF-15, respectively, while the final setting time increased from 120 to 160, 220, and 265 min. A comparable trend was observed for the TF series, with the initial setting time reaching 120, 160, and 180 min, and the final setting time reaching 180, 230, and 250 min, respectively. It can be observed that the use of TF in the composition can prolong the initial setting time up to 2.2 times, compared to the control paste [64]. This behavior indicates that partial cement replacement with TF made the early structure formation process more visible, which can be regarded as a controlled modification of hydration development rather than an adverse effect.

The influence became more pronounced in the presence of chemical admixtures. In the PC-modified pastes, the initial setting time was 210, 180, and 450 min for PC dosages of 0.5, 1.0, and 1.5%, respectively, whereas the final setting time increased to 280, 410, and 610 min, respectively. Although the initial setting time at 1.0% PC was slightly lower than at 0.5%, the overall tendency indicates that increasing PC dosage shifted the setting process to later times, thereby providing a broader time window for dispersion, structural rearrangement, and fresh-state workability [40,65]. The AE-containing systems also exhibited extended setting times, with initial setting times of 240, 260, and 300 min and final setting times of 340, 340, and 390 min for AE dosages of 0.15, 0.30, and 0.45%, respectively. Thus, while AE had only a limited effect on paste spread, it noticeably influenced the kinetics of setting and contributed to a more gradual development of the fresh cement pastes [66].

The combined use of PC and AE resulted in the most substantial change in setting behavior, indicating a synergistic influence of the two admixtures on early-age processes. The initial setting times increased to 260, 370, and 450 min for PC-AE-15, PC-AE-30, and PC-AE-45, respectively, while the corresponding final setting times reached 510, 550, and 610 min, respectively. The combined effect of both admixtures prolongs the initial setting time by up to 2.5 times compared with the PC-only paste at the same PC dosage, and by up to 1.5 times compared with the AE-only pastes [67].

A similarly pronounced effect was observed in the ternary fly ash-containing systems: UF-PC-AE showed initial and final setting times of 270 and 520 min, whereas TF-PC-AE showed values of 240 and 500 min, respectively. Overall, the obtained results confirm that the combined use of mineral and chemical modifiers provides an effective tool for controlling the early-age behavior of cement systems, allowing the setting process to be adjusted in a targeted manner in accordance with technological requirements.

**Figure 8:**
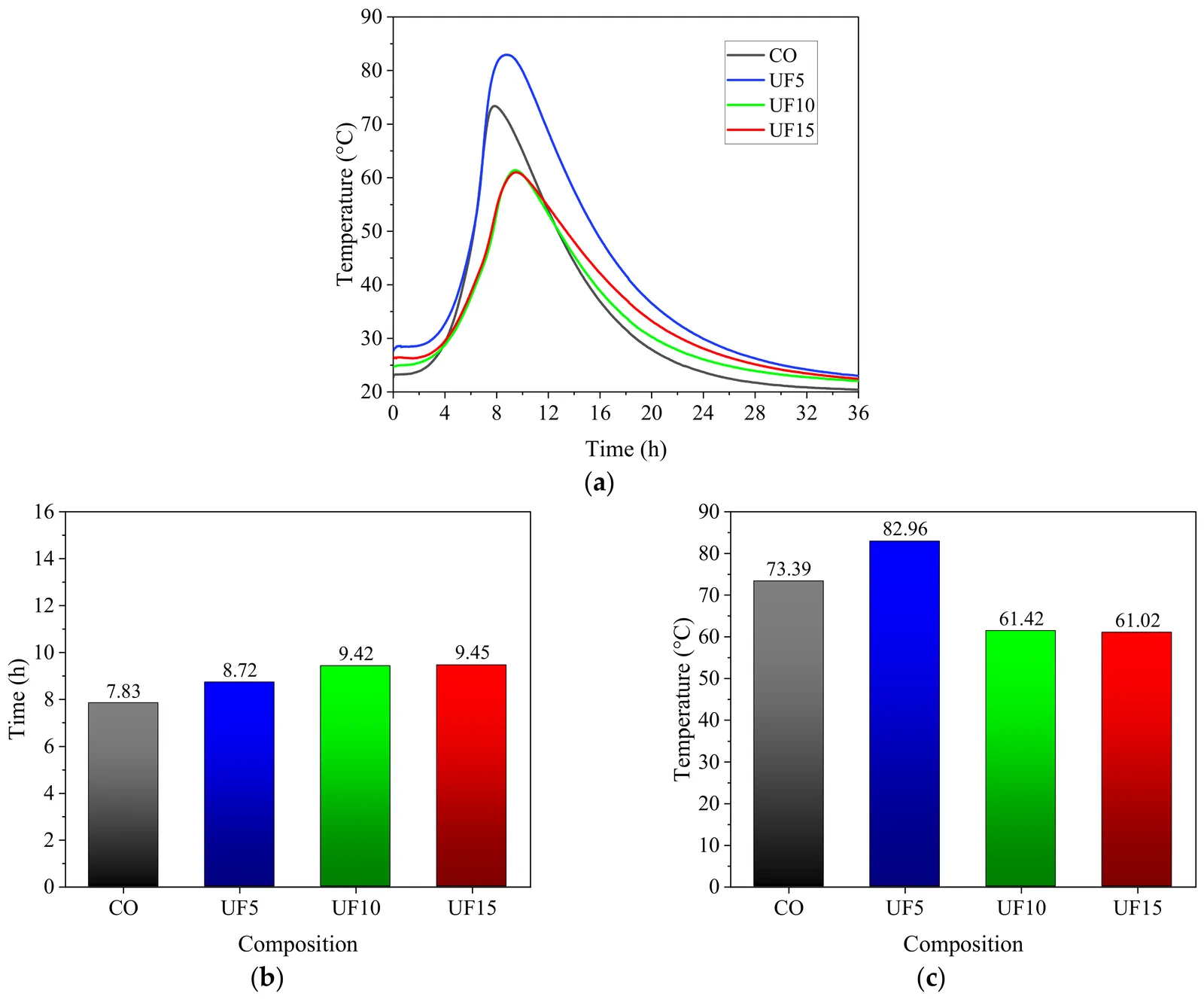
Early hydration behavior of cement pastes containing different UF contents: (**a**) exothermic curves over 36 h; (**b**) time to reach maximum temperature; (**c**) maximum hydration temperature.

To further clarify the effect of mineral and chemical admixtures on the early hydration process, the temperature evolution of cement pastes was monitored during the first 36 h, and the corresponding exothermic curves and the time to reach the maximum exothermic temperature, corresponding to the peak of hydration, are presented in Figure 8. These parameters provide additional insight into the kinetics of hydration and complement the previously discussed results on fresh-state behavior and setting time.

As shown in Figure 8, the incorporation of UF in the composition markedly affected the exothermic temperature response of the cement pastes. The reference composition reached its maximum temperature of 73.39 °C after 7.83 h, whereas the mixture in which cement was replaced by 5% UF exhibited a higher peak temperature of 82.96 °C, reached after 8.72 h. This result suggests that a low UF dosage promoted a more intense early hydration process, likely due to a filler effect and improved particle packing, which enhanced nucleation of hydration products [46,68]. However, with further increases in UF replacement to 10% and 15%, the maximum temperature decreased markedly to 61.42 °C and 61.02 °C, respectively, while the time to peak increased to 9.42 h and 9.45 h, respectively. That indicates that higher replacement levels reduced the intensity of early hydration due to clinker mineral dilution and the relatively low reactivity of UF at the initial stage.

**Figure 9:**
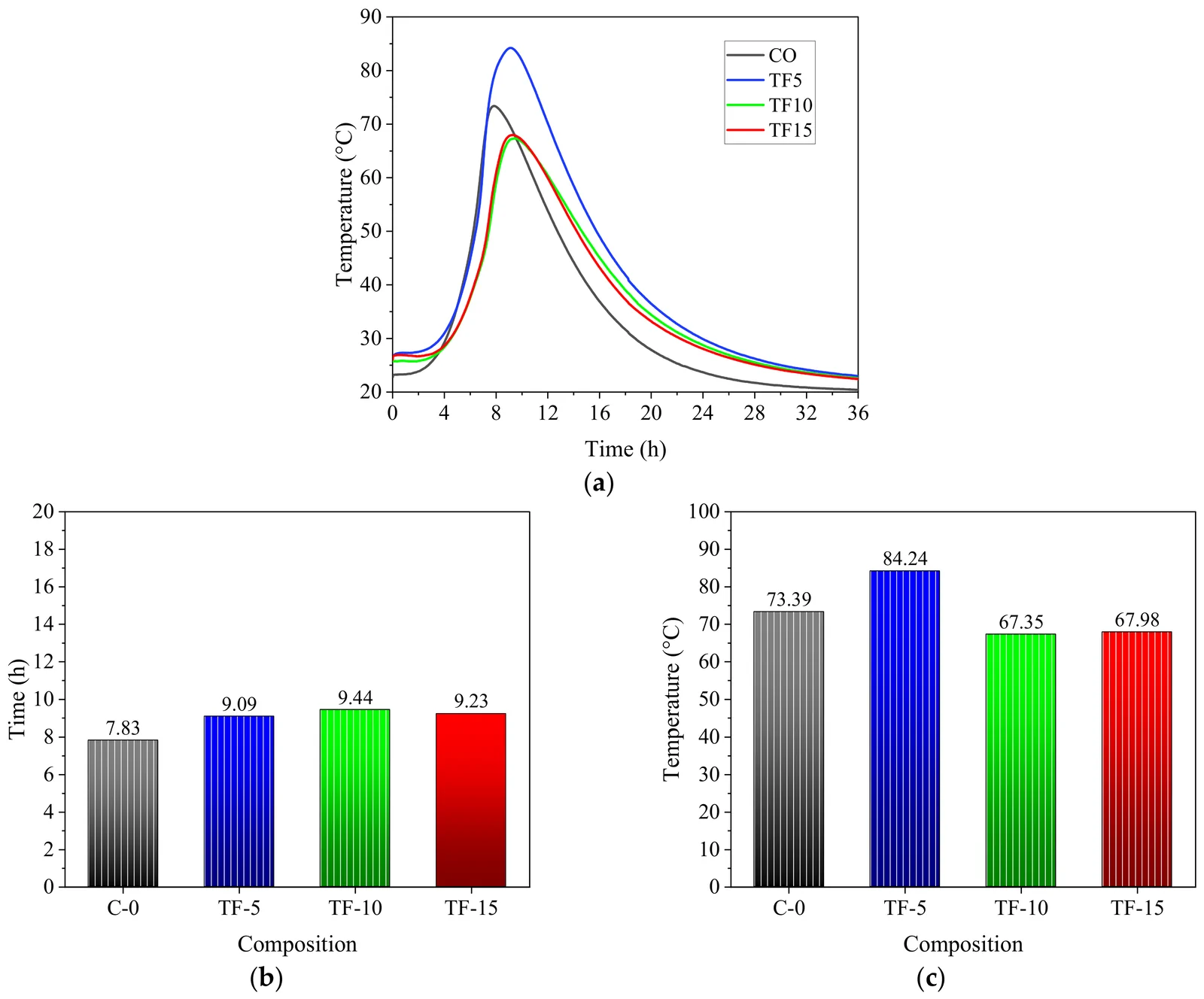
Early hydration behavior of cement pastes containing different TF contents: (**a**) exothermic curves over 36 h; (**b**) time to reach maximum temperature; (**c**) maximum hydration temperature.

A similar result, with a more pronounced beneficial effect at low dosage, was observed for the TF-containing mixtures (Figure 9). The paste containing 5% TF reached the highest temperature among all TF-containing pastes, namely 84.24 °C, after 9.09 h, exceeding not only the control but also the corresponding UF mixture.

This behavior confirms that mechanical activation enhanced the reactivity of TF and intensified early hydration, most likely through the combined effects of increased fineness, higher surface area, and accelerated hydrate nucleation [69]. This agrees with our earlier study of the same mechanically activated ash [32], in which grinding accelerated the early hydration of cement pastes and increased the exothermic response as a result of the finer particle size and the enhanced dissolution of the activated glass phase. At the same time, increasing the TF content to 10% and 15% reduced the peak temperature to 67.35 °C and 67.98 °C, respectively, with the time to peak remaining around 9.23–9.44 h. Thus, although TF performed better than UF, excessive replacement still reduced the thermal effect due to the lower proportion of cement clinker in the system.

**Figure 10:**
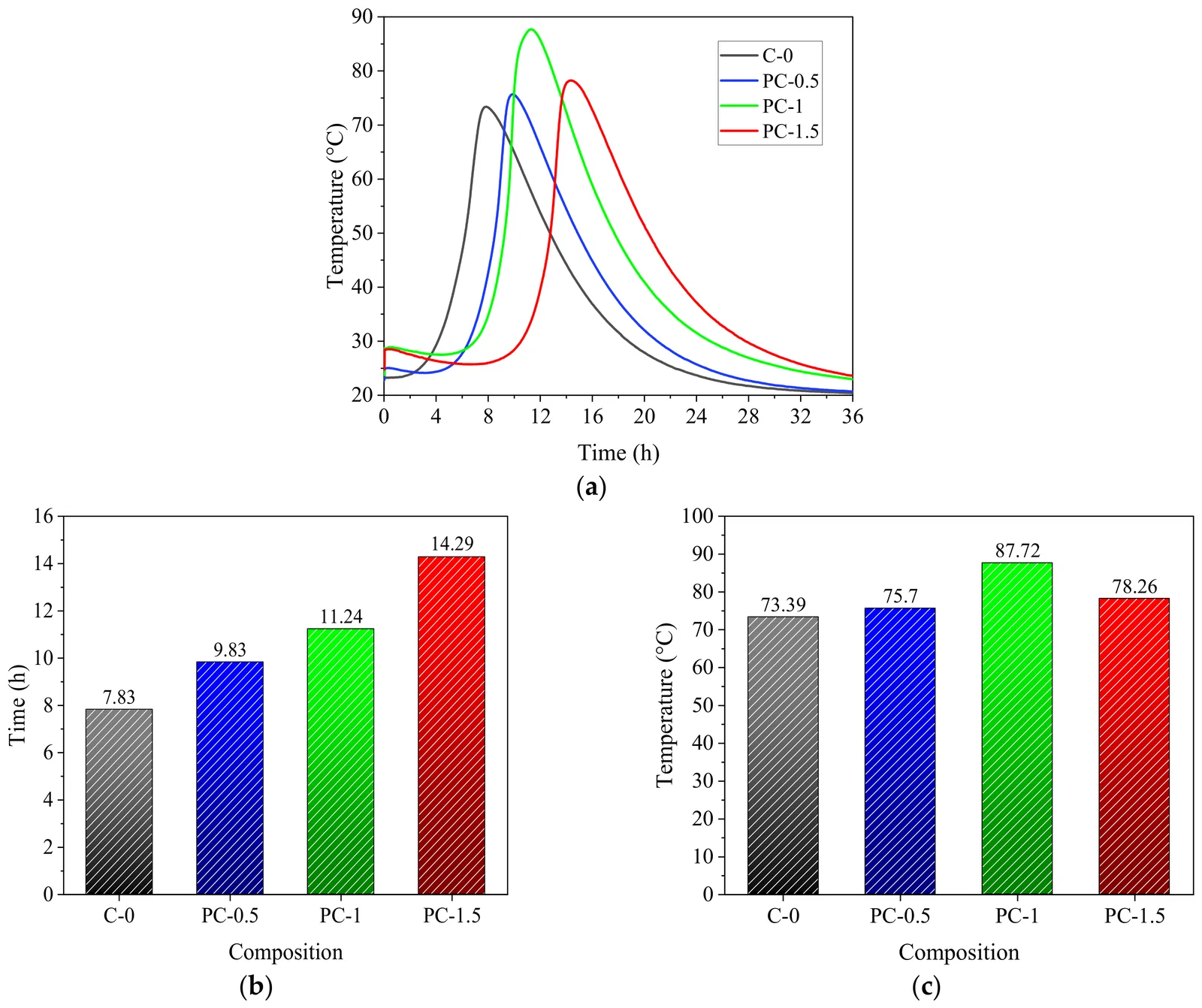
Early hydration behavior of cement pastes containing different PC dosages: (**a**) exothermic curves over 36 h; (**b**) time to reach maximum temperature; (**c**) maximum hydration temperature.

The effect of the PC on hydration kinetics is presented in Figure 10. All PC-modified pastes showed a delay in reaching the EXO maximum temperature relative to the control paste, confirming the retarding effect of the admixture on early hydration. The control mixture reached the maximum temperature after 7.83 h, while the mixtures containing 0.5%, 1.0%, and 1.5% PC reached the peak after 9.83 h, 11.24 h, and 14.29 h, respectively. Despite this retardation, the mixture containing 1.0% PC exhibited the highest maximum temperature of 87.72 °C, the highest among all tested compositions. This result indicates that a moderate PC dosage delayed the hydration peak but did not suppress the hydration process; rather, it appears to have promoted a more intense heat release once hydration was initiated, likely due to improved particle dispersion and more efficient wetting of cement grains. At a dosage of 1.5%, however, the peak temperature decreased to 78.26 °C, suggesting that excessive adsorption of PC on the cement particles led to stronger retardation and partial limitation of early hydration intensity [70]. This dual behavior reflects the two competing actions of the polycarboxylate ether: its adsorption on the cement grains retards C_3_S dissolution and C–S–H nucleation, shifting the peak to a later time, while its dispersing effect deflocculates the grains and releases entrapped water, so that once hydration starts it proceeds over a larger wetted surface and releases heat more intensely, raising the peak. Only at the highest dosage of 1.5% PC does the retarding action outweigh the dispersing benefit, lowering both the reaction rate and the peak temperature.

**Figure 11:**
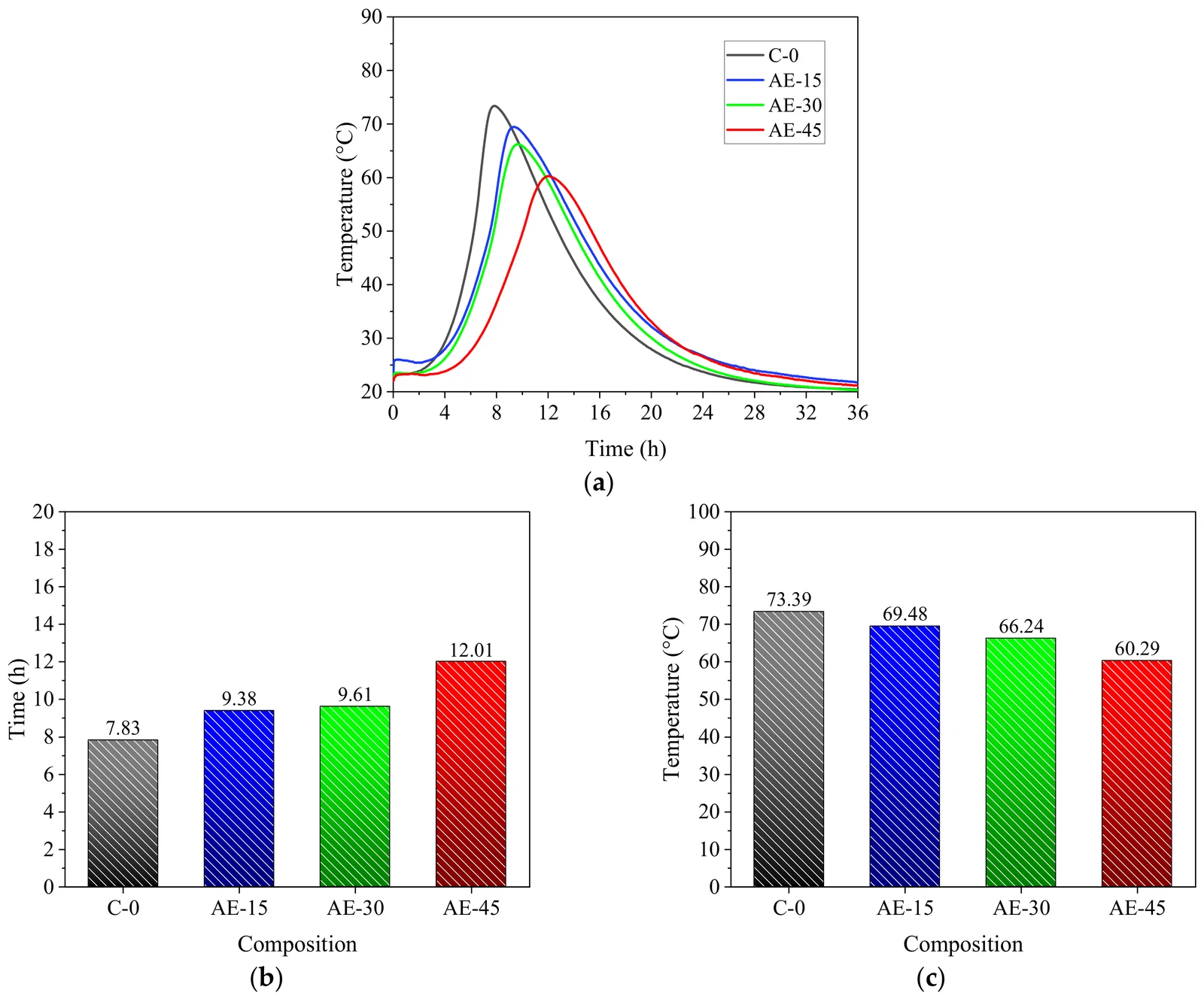
Early hydration behavior of cement pastes containing different AE dosages: (**a**) exothermic curves over 36 h; (**b**) time to reach maximum temperature; (**c**) maximum hydration temperature.

The incorporation of the AE produced a different hydration pattern (Figure 11). In all AE-containing pastes, the maximum hydration temperature decreased progressively with increasing dosage, while the time to reach the temperature maximum peak increased. More specifically, the maximum temperature dropped to 69.48 °C, 66.24 °C, and 60.29 °C for 0.15%, 0.30%, and 0.45% AE, respectively, whereas the corresponding time to peak increased to 9.38 h, 9.61 h, and 12.01 h, respectively. These results indicate that AE consistently slowed down the early hydration process and reduced the intensity of heat release. This behavior can be attributed to the formation of a more discontinuous liquid-solid contact environment and the entrainment of air voids, which may reduce the rate of hydration reactions in the early stage. This is consistent with the adsorption of the air-entraining surfactant onto the cement particles, which slows early hydration [66].

**Figure 12:**
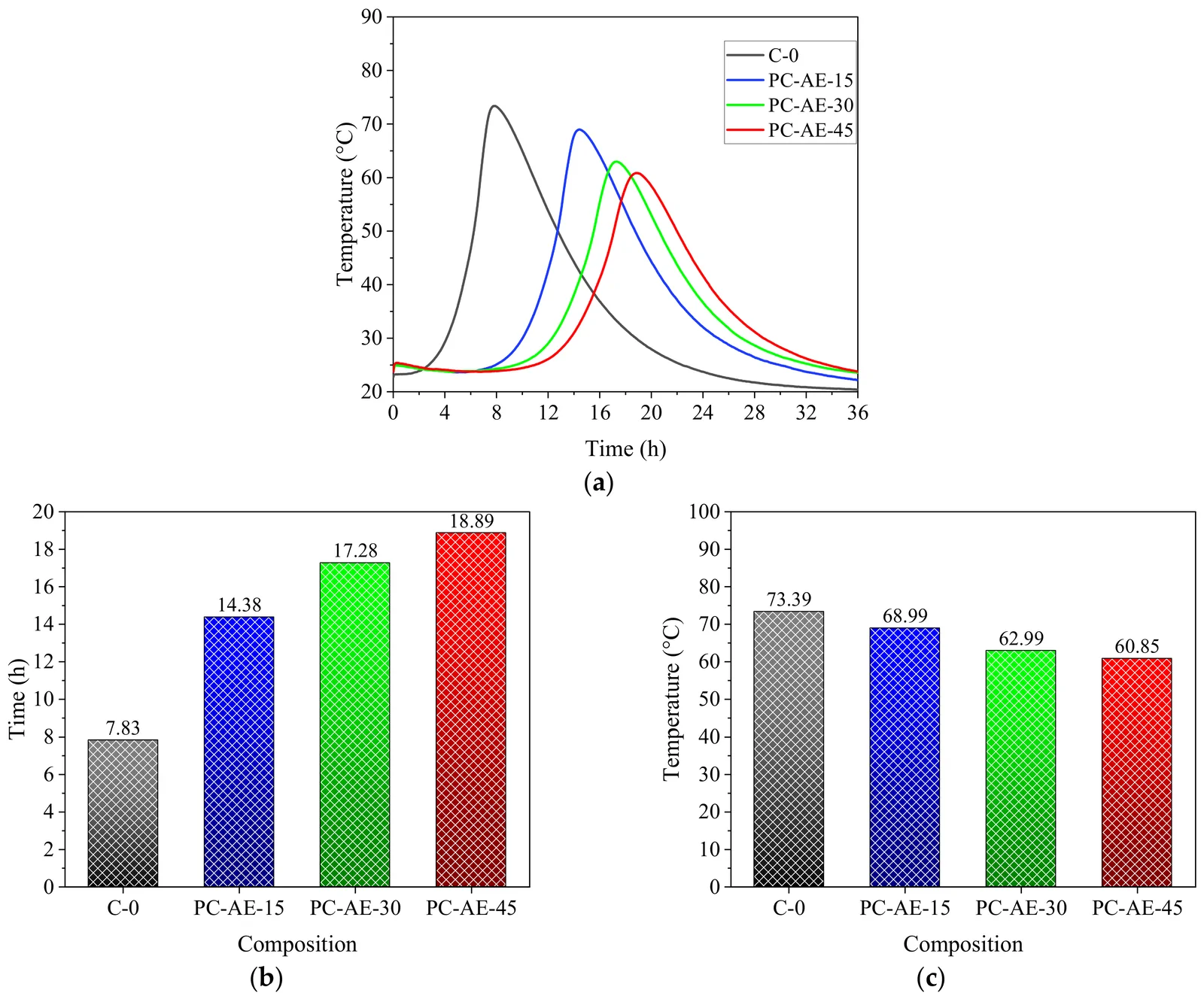
Early hydration behavior of cement pastes containing PC and different AE dosages: (**a**) exothermic curves over 36 h; (**b**) time to reach maximum temperature; (**c**) maximum hydration temperature.

The combined use of PC (1%) and AE further enhanced the retardation of the hydration process (Figure 12). Compared with the control and the individual PC-modified pastes, the pastes with binary admixture systems reached the EXO maximum temperature considerably later, namely at 14.38 h, 17.28 h, and 18.89 h for PC-AE-15, PC-AE-30, and PC-AE-45, respectively. Compared to PC-1 paste, combining PC with AE increases the time to reach the EXO peak in pastes by 22%, 35%, and 40.6%. Such retardation is typical of polycarboxylate superplasticizers adsorbing on cement grains [65].

At the same time, the maximum peak temperature of the EXO decreased to 68.99 °C, 62.99 °C, and 60.85 °C, compared with the PC-1 paste data, representing 21.3%, 28.2%, and 30.62%, respectively.

That demonstrates that the combined action of PC and AE admixtures substantially prolonged the induction period and lowered the thermal effect of hydration. The results are in good agreement with the previously observed extension of setting times in these compositions.

**Figure 13:**
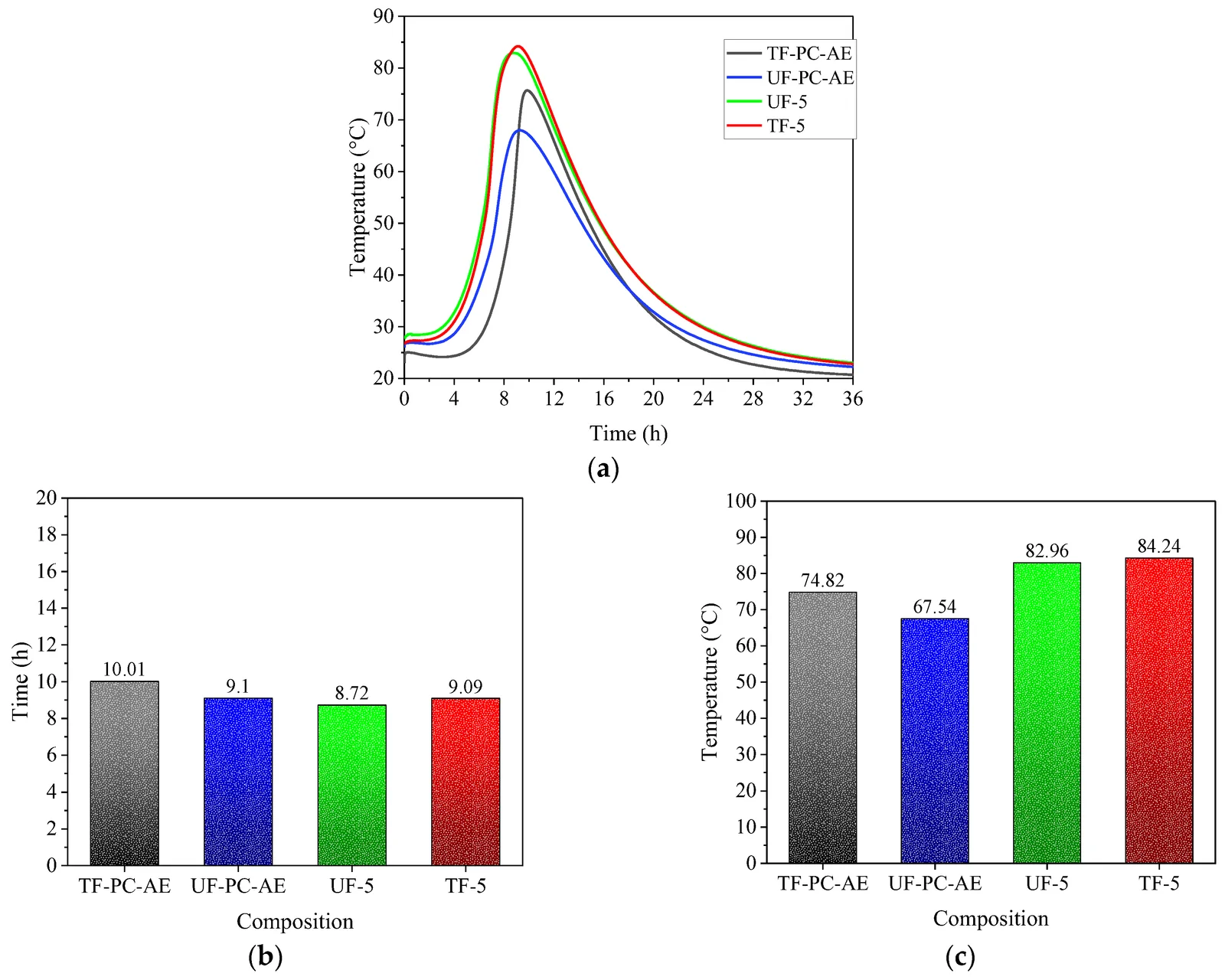
Early hydration behavior of cement pastes containing the same amount of UF-5 and TF-5, TF-PC-AE, and UF-PC-AE: (**a**) exothermic curves over 36 h; (**b**) time to reach maximum temperature; (**c**) maximum hydration temperature.

A comparison of the complex multicomponent systems is shown in Figure 13. The composition TF-PC-AE reached a maximum temperature of 74.82 °C after 10.01 h, while the reference composition TF-5 exhibited a maximum temperature 11.2% higher and reached its EXO maximum 10.1% earlier. The composition UF-PC-AE reached its EXO maximum at 67.54 °C after 9.1 h, whereas the reference composition UF-5 showed a maximum temperature 18.52% higher and reached the peak 4.4% earlier. Relative to the single fly ash compositions TF-5 and UF-5, both combined admixture pastes exhibited delayed hydration parameters; however, the TF-containing paste preserved a higher thermal peak than the corresponding UF-containing paste. That confirms that mechanical activation of fly ash partially compensated for the retarding effect caused by the combined chemical admixtures and contributed to a more intense early hydration process. Therefore, among the complex modified pastes, mechanically activated fly ash appears more favorable for maintaining hydration activity during the first 24 h. This agrees with calorimetry studies showing that mechanical activation accelerates the fly-ash–cement reaction [69].

Overall, the exothermic analysis demonstrated that the early hydration behavior of cement pastes was strongly dependent on both the type and dosage of the admixtures. Low dosages of fly ash, especially mechanically activated fly ash, enhanced the hydration parameters and increased the EXO peak temperature relative to the corresponding single fly ash compositions UF-5 and TF-5. In contrast, higher replacement levels reduced the thermal effect by diluting clinker minerals [68]. PC delayed the hydration peak but, at the optimal dosage of 1%, led to the highest EXO maximum temperature among all tested compositions, indicating a more efficient, intensified hydration process due to improved dispersion of cement particles. In contrast, AE and especially the combined PC–AE admixture system reduced the peak temperature and considerably prolonged the time to maximum heat evolution, confirming their retarding effect on early hydration. These findings are consistent with the setting time results and demonstrate that the hydration kinetics of the investigated cement-based pastes can be effectively controlled by selecting an appropriate combination of mineral additives and chemical admixtures.

### 3.2. Microstructural and Phase Analysis

To gain deeper insight into the effects of UF, TF, and AE admixtures on the structure-forming processes, a microstructural and phase analysis of hydrated cement paste samples was performed. These sand-free paste compositions correspond to the mix designs given in Table 3, namely the reference paste (C-0) and the pastes containing untreated fly ash (UF), mechanically activated fly ash (TF), and the air-entraining admixture (AE). The microstructural features were evaluated by scanning electron microscopy (SEM), whereas the phase composition of the modified systems was examined by X-ray diffraction (XRD). This combined approach enabled correlating the morphological features of the hydrated cement matrix with the phase evolution observed in the presence of different admixtures.

**Figure 14:**
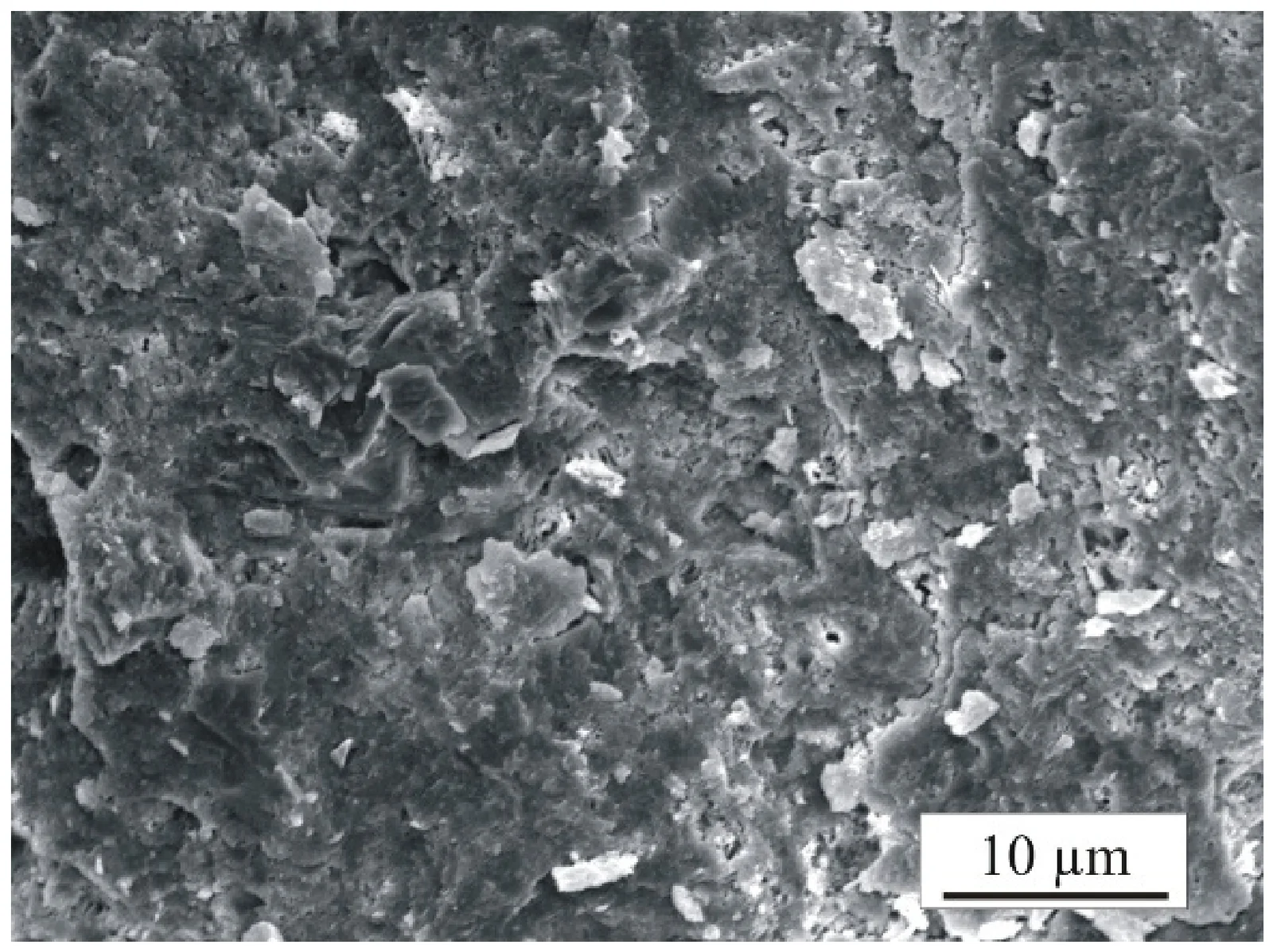
SEM microstructure of the control hydrated cement paste sample (C-0).

**Figure 15:**
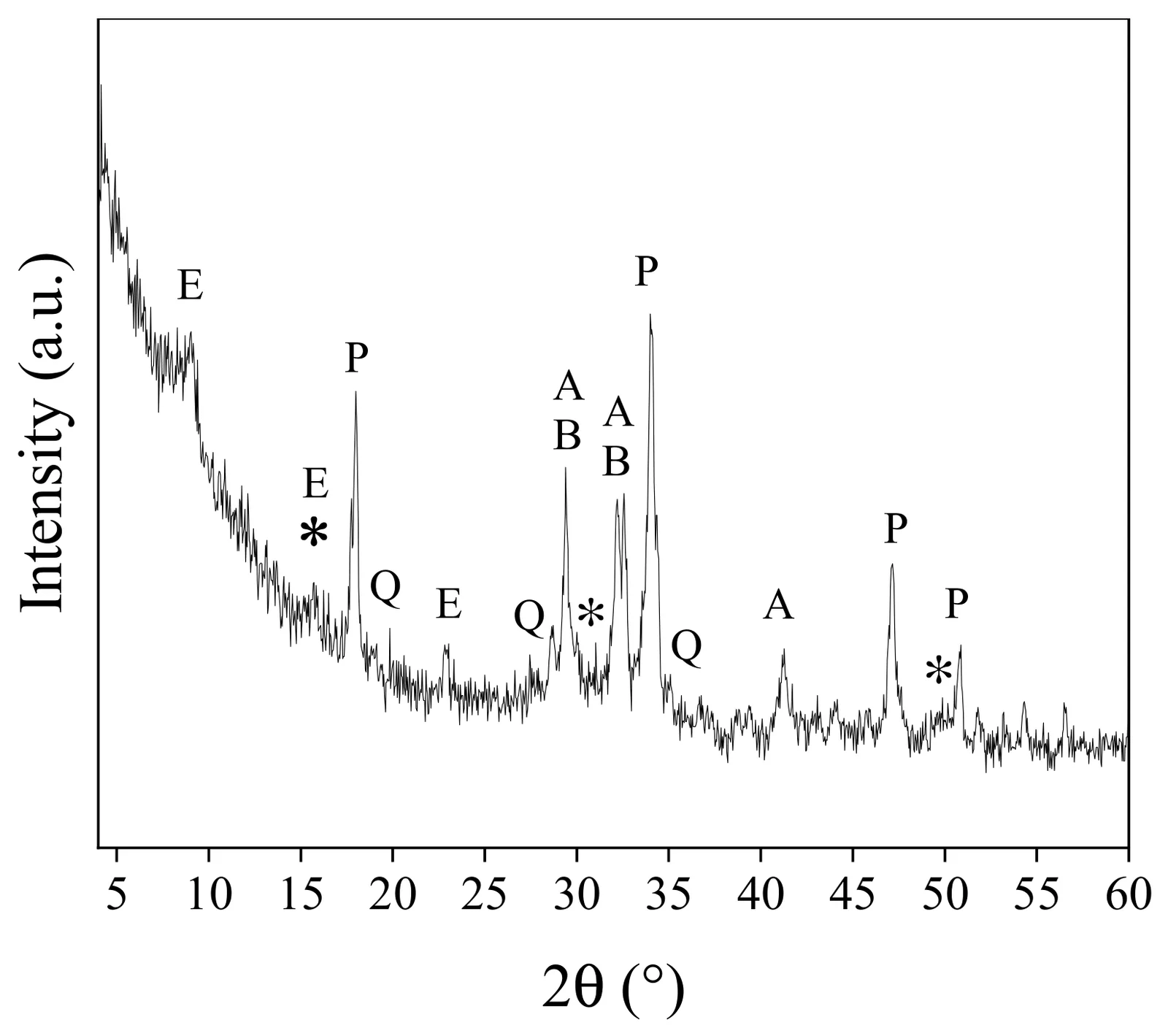
X-ray diffraction pattern of the hydrated cement paste sample (C-0). E—Ettringite; P—Portlandite; Q—Quartz; B—Belite; A—Alite; *—Calcium silicate hydrates.

The SEM image of the control hydrated cement paste (Figure 14) reveals a relatively dense matrix with scattered pores distributed throughout the microstructure. The overall morphology is homogeneous, although isolated microvoids and localized non-uniformity in particle distribution are occasionally observed, which is typical of unmodified cement paste. This composition served as the reference for evaluating microstructural changes induced by the incorporation of fly ash and chemical admixtures. According to the XRD results for the control hydrated cement paste (Figure 15), the main phases typical of a hydrated cement paste are observed: portlandite Ca(OH)2, ettringite, calcium silicate hydrates (C–S–H), quartz, and residual clinker minerals.

**Figure 16:**
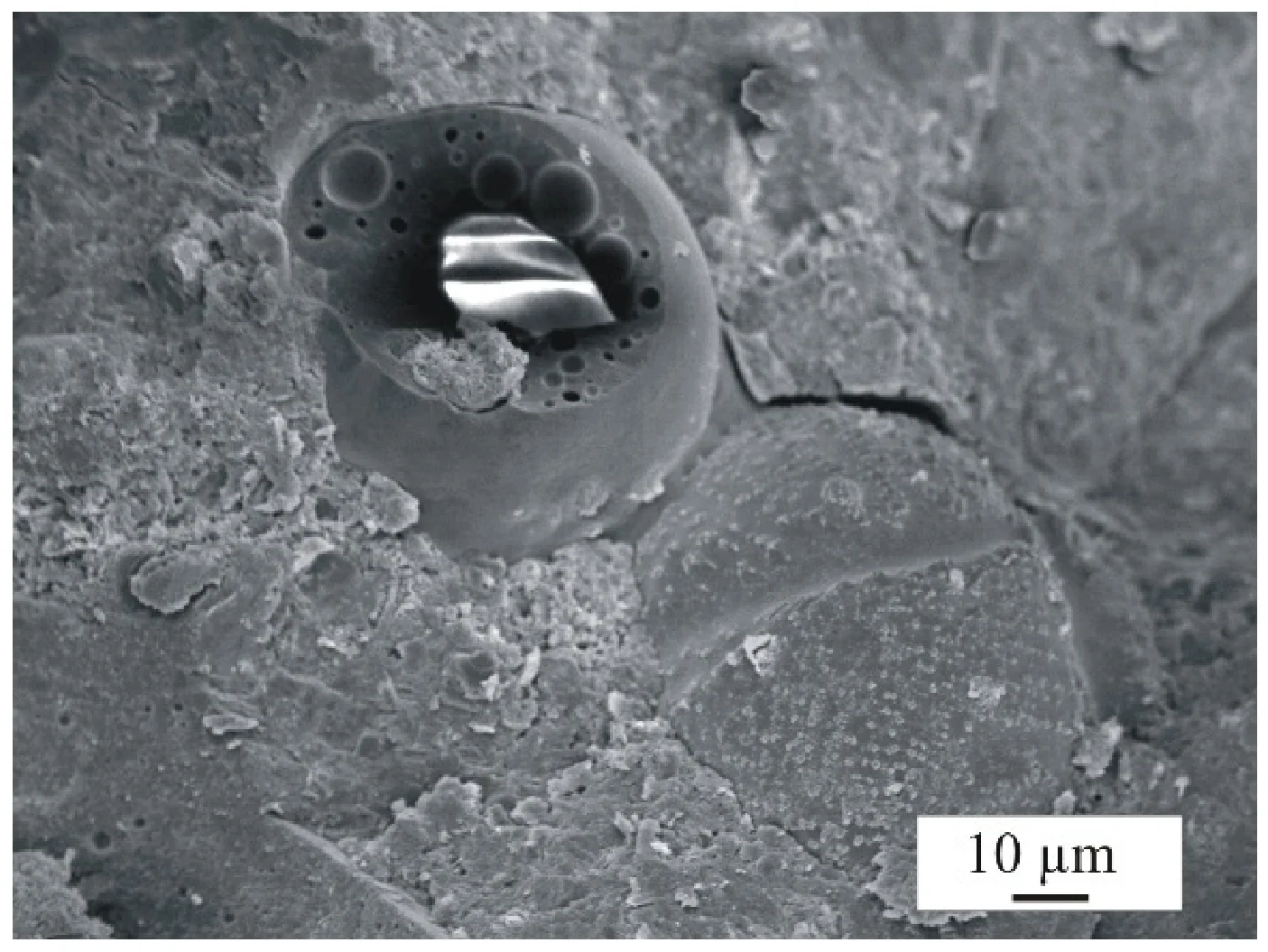
SEM microstructure of the hydrated cement paste containing untreated fly ash (UF).

The SEM image of the composition containing UF (Figure 16) clearly shows relatively coarse ash particles that largely preserve their original morphological features. These inclusions are not fully uniformly distributed within the hydrated cement matrix, and their degree of structural integration remains limited. That suggests that, at this stage, UF mainly acts as a mineral microfiller rather than being actively involved in secondary structure formation. The presence of relatively large ash particles indicates a lower degree of reactive participation compared with mechanically activated TF.

**Figure 17:**
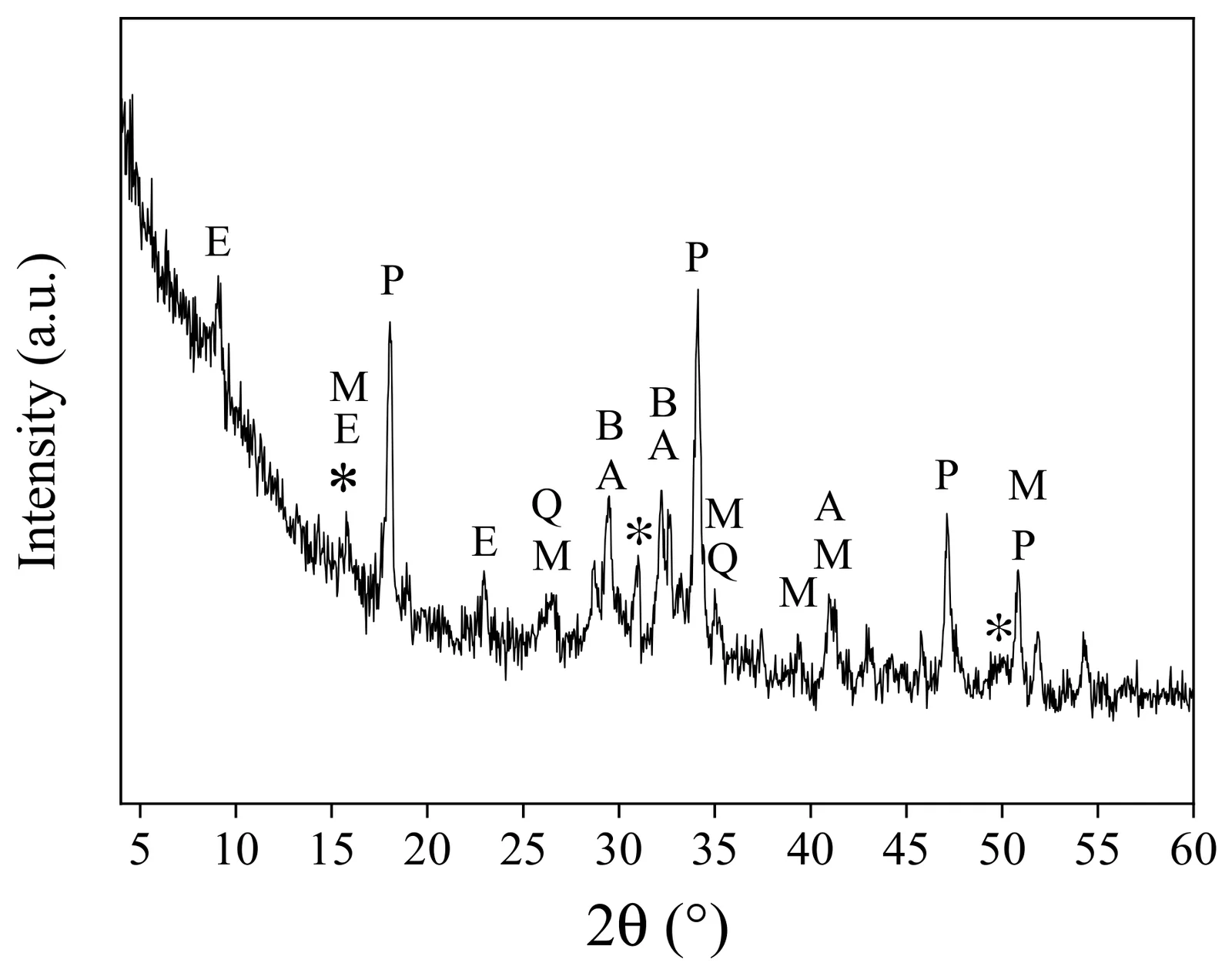
X-ray diffraction pattern of the hydrated cement paste containing untreated fly ash (UF). E—Ettringite; M—Mullite; P—Portlandite; Q—Quartz; B—Belite; A—Alite; *—Calcium silicate hydrates.

According to the XRD results for the composition containing untreated fly ash (Figure 17), the sample contains the main phases typical of a hydrated cement paste modified with a fly ash [33], including calcium silicate hydrates (C–S–H), ettringite, portlandite Ca(OH)_2_, quartz, mullite, and residual clinker minerals. The presence of mullite indicates the mineral nature of fly ash, whereas portlandite, ettringite, and calcium silicate hydrates reflect the ongoing cement hydration process. The identified phase assemblage indicates that UF retains the characteristics of the original mineral component while also being incorporated into the developing cementitious structure.

**Figure 18:**
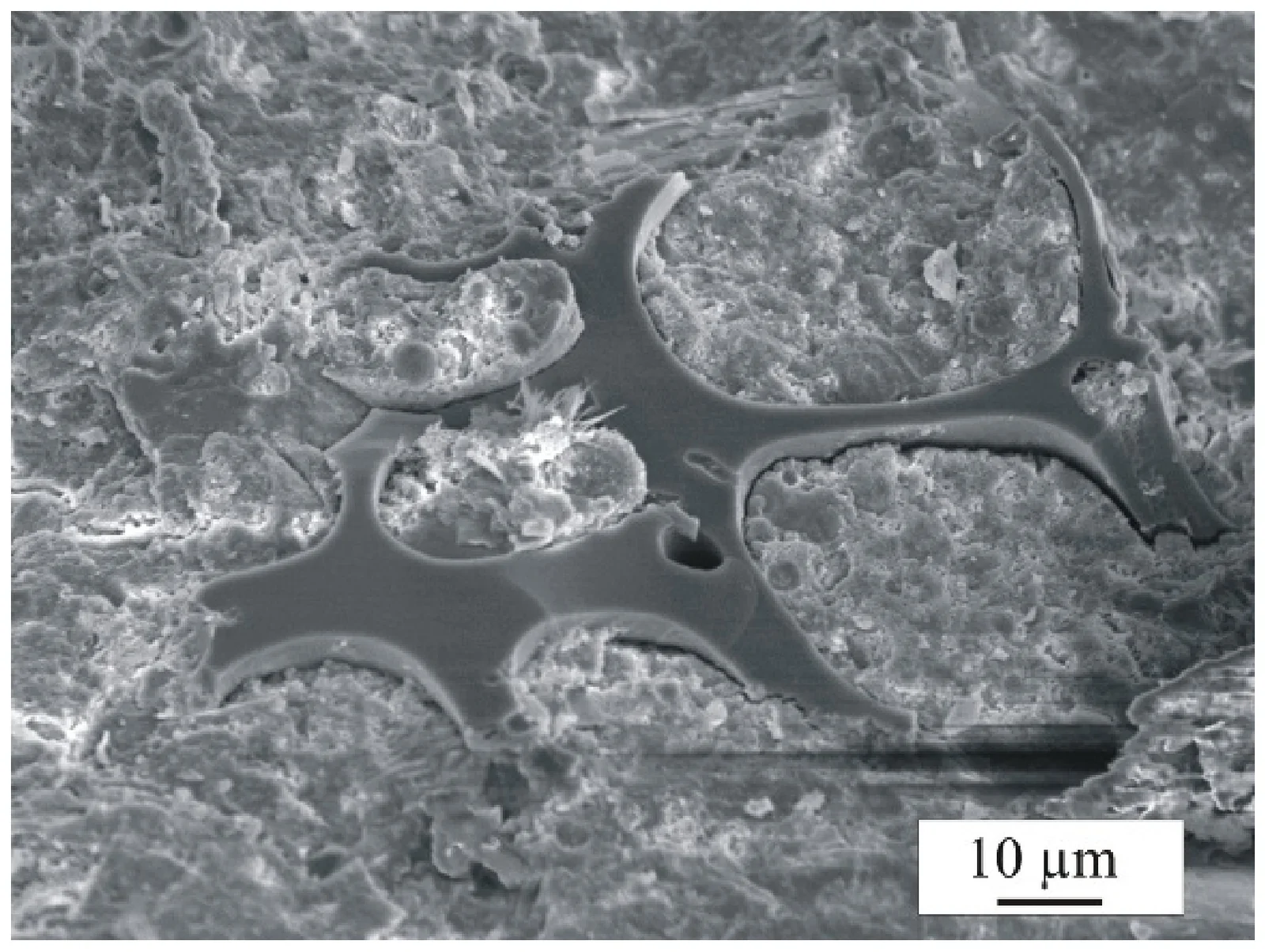
SEM microstructure of the hydrated cement paste containing mechanically activated fly ash (TF).

The SEM image of the composition containing TF (Figure 18) shows finer ash fragments distributed more uniformly, and the structure is visibly denser throughout the hydrated cement matrix. In several areas, pores partially filled with hydration products can be observed, and hydration products are also seen growing along the pore walls. Compared with the composition containing UF, the structure appears more homogeneous. That is likely associated with the increased specific surface area and higher reactivity of the TF after mechanical activation, which promotes its more active participation in the structure-forming processes.

**Figure 19:**
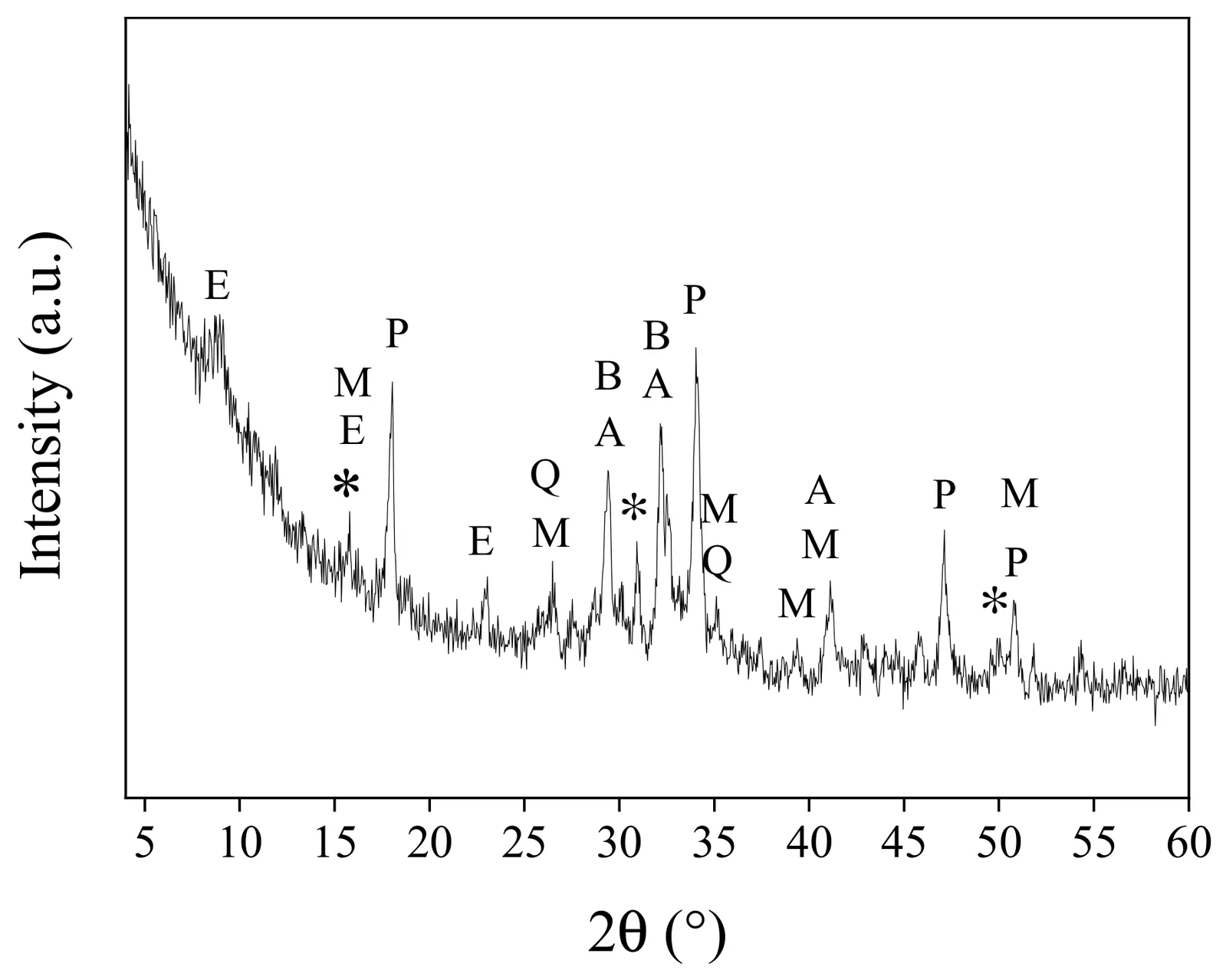
X-ray diffraction pattern of the hydrated cement paste containing mechanically activated TF. E—Ettringite; M—Mullite; P—Portlandite; Q—Quartz; B—Belite; A—Alite; *—Calcium silicate hydrates.

The XRD pattern of the hydrated cement paste containing mechanically activated fly ash (Figure 19) confirms the presence of the main phases typical of a hydrated cement system, including C–S–H, portlandite Ca(OH)_2_, quartz (Q), mullite (M), and residual clinker phases such as C_2_S and C_3_S. The XRD pattern suggests the formation of hydration products, while the broader amorphous background may be associated with C–S–H and the amorphous fraction of fly ash [32]. Additionally, it is worth mentioning that, according to published research [71,72,73], anionic polymers such as PCE have been shown to interact with dissolving silicates and aluminates, form complexes, and bind with cement hydroxides to form layered double hydroxides or C–S–H, thereby contributing to more complete cement hydration. In a study [74], electrical conductivity measurements showed that PCE inhibits the dissolution of C_3_S and slows the increase in electrical conductivity. It is thought to promote the formation of C–S–H and other hydrates, with complexes formed between Ca^2+^ ions and high-charge-density carboxylate polymers during the hydration of C_3_S and the formation of C–S–H nuclei [75]. The presence of hydration products is consistent with the development of the cementitious matrix. These results indicate that the incorporation of mechanically activated TF affects the development of the hydrated cement matrix while preserving the principal phase assemblage characteristic of fly ash-modified cement paste.

The identified phases can be related to the pozzolanic reaction between the amorphous aluminosilicate part of the ash and the portlandite formed during clinker hydration (Equations (3) and (4)):

Ca(OH)_2_ + SiO_2_ (amorphous) + nH_2_O → C–S–H (3)

Ca(OH)_2_ + Al_2_O_3_ (amorphous) + SiO_2_ + nH_2_O → C–A–S–H (4)

After mechanical activation this reaction proceeds more intensively. The glassy spheres are broken into angular fragments, so a larger surface of the ash is in contact with the alkaline pore solution. In addition, grinding introduces structural disorder into the glass, which is reflected in the reduced crystallinity of TF; the weakened Si–O–Si and Si–O–Al bonds are more easily attacked by OH^−^ ions, and silicate and aluminate species pass into the pore solution faster. As a result, more Ca(OH)_2_ is consumed and additional C–S–H is formed. That is in agreement with the broader amorphous background in the TF pattern (Figure 19) and with the denser and more homogeneous matrix observed by SEM (Figure 18).

**Figure 20:**
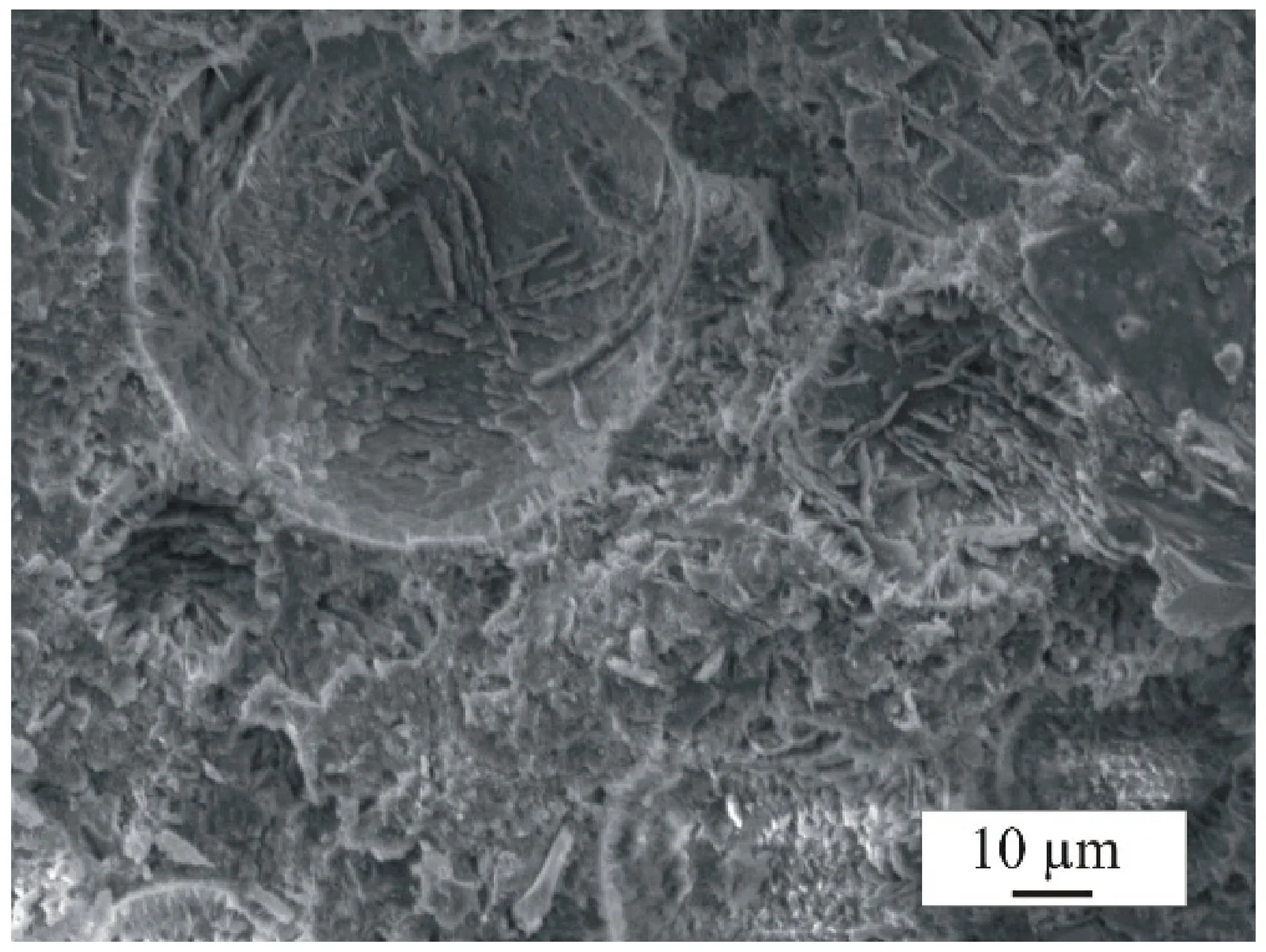
SEM microstructure of the hydrated cement paste containing AE.

The SEM image of the hydrated cement paste containing AE (Figure 20) reveals the presence of relatively large and well-defined pores within the cementitious matrix. Hydration products can be observed growing inside these pores and along their walls, indicating the development of the hydrated structure around the entrained air voids. Such a pore system is characteristic of air-entrained cement-based materials and may improve freeze–thaw resistance by providing pressure-relief space during water freezing.

**Figure 21:**
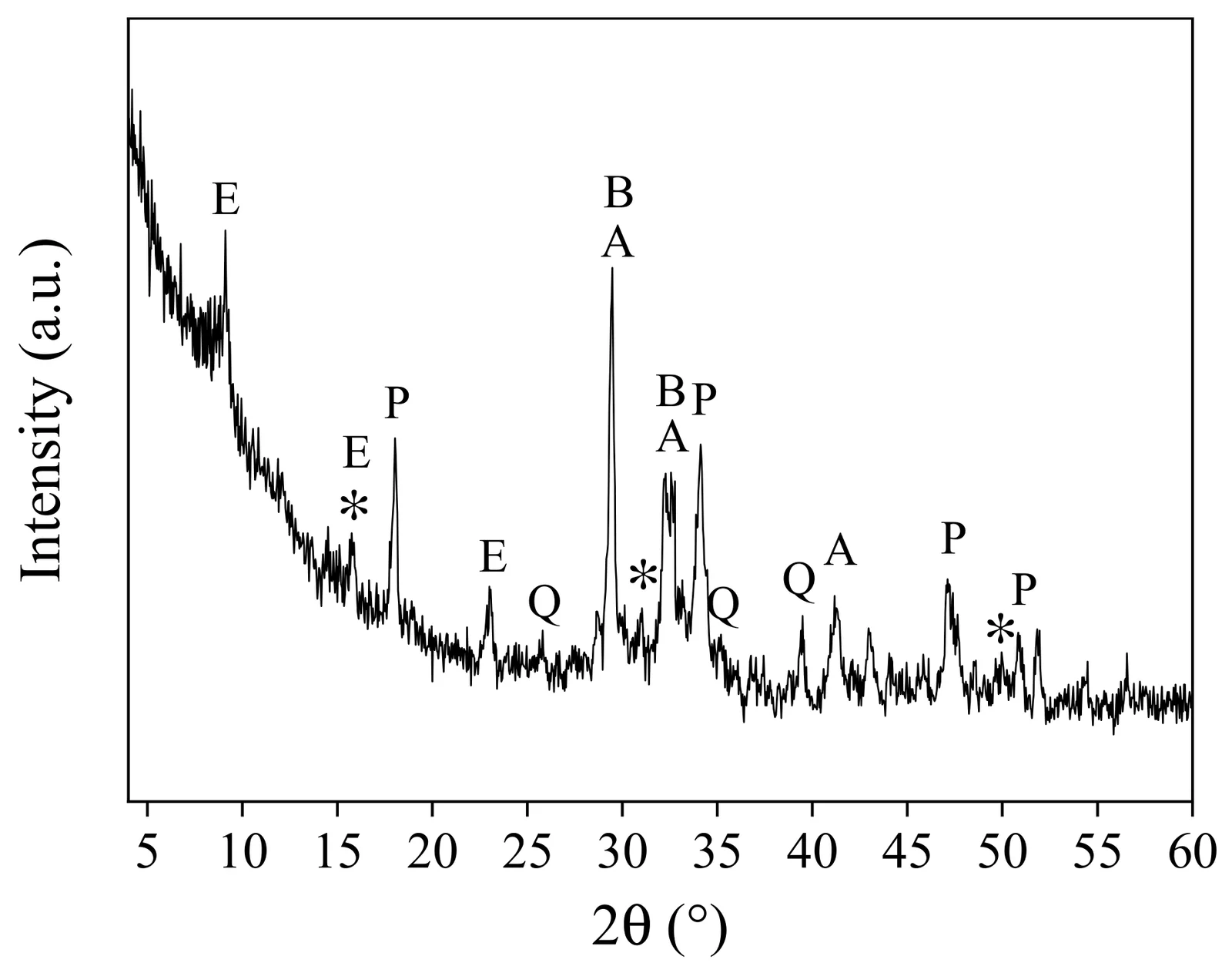
X-ray diffraction pattern of the hydrated cement paste containing AE. E—Ettringite; P—Portlandite; Q—Quartz; B—Belite; A—Alite; *—Calcium silicate hydrates.

The XRD pattern of the composition containing AE (Figure 21) shows that the phase composition generally remains typical of an ordinary hydrated cement paste, including ettringite, portlandite, and unreacted cement minerals. It can be observed that the intensities of the diffraction peaks of portlandite and ettringite are more pronounced in AE-containing pastes. It is assumed that in the formed pores, due to sufficient pore space, more active hydration of cement minerals occurs.

That indicates that the principal effect of AE is manifested primarily through modifications in pore structure and pore volume, rather than through a substantial change in the crystalline phase assemblage. Accordingly, the XRD data for this composition should be interpreted together with the SEM observations, which confirm the development of a more pronounced air-void system.

Overall, the results of the microstructural and X-ray diffraction analyses showed that the investigated modifiers affect the structure-forming processes in hydrated cement paste in different ways. UF largely preserves its original morphological and mineralogical characteristics and exhibits only limited interaction with the hydrated cement matrix. In contrast, mechanical activation promotes a more uniform particle distribution and enhances TF participation in the formation of the cementitious structure. The AE forms a more developed pore system that may be beneficial for the crystallization of hydration products and, at the same time, improve resistance to cyclic freezing and thawing. This contrast shows how the pozzolanic reactivity of the ash can be enhanced: milling shatters the smooth glassy UF spheres into finer angular fragments with a higher specific surface area and fresh defect-rich surfaces [52], exposing more reactive glass and creating additional nucleation sites, which accelerates portlandite consumption and secondary C–S–H formation [35].

### 3.3. Water Absorption and Density

**Figure 22:**
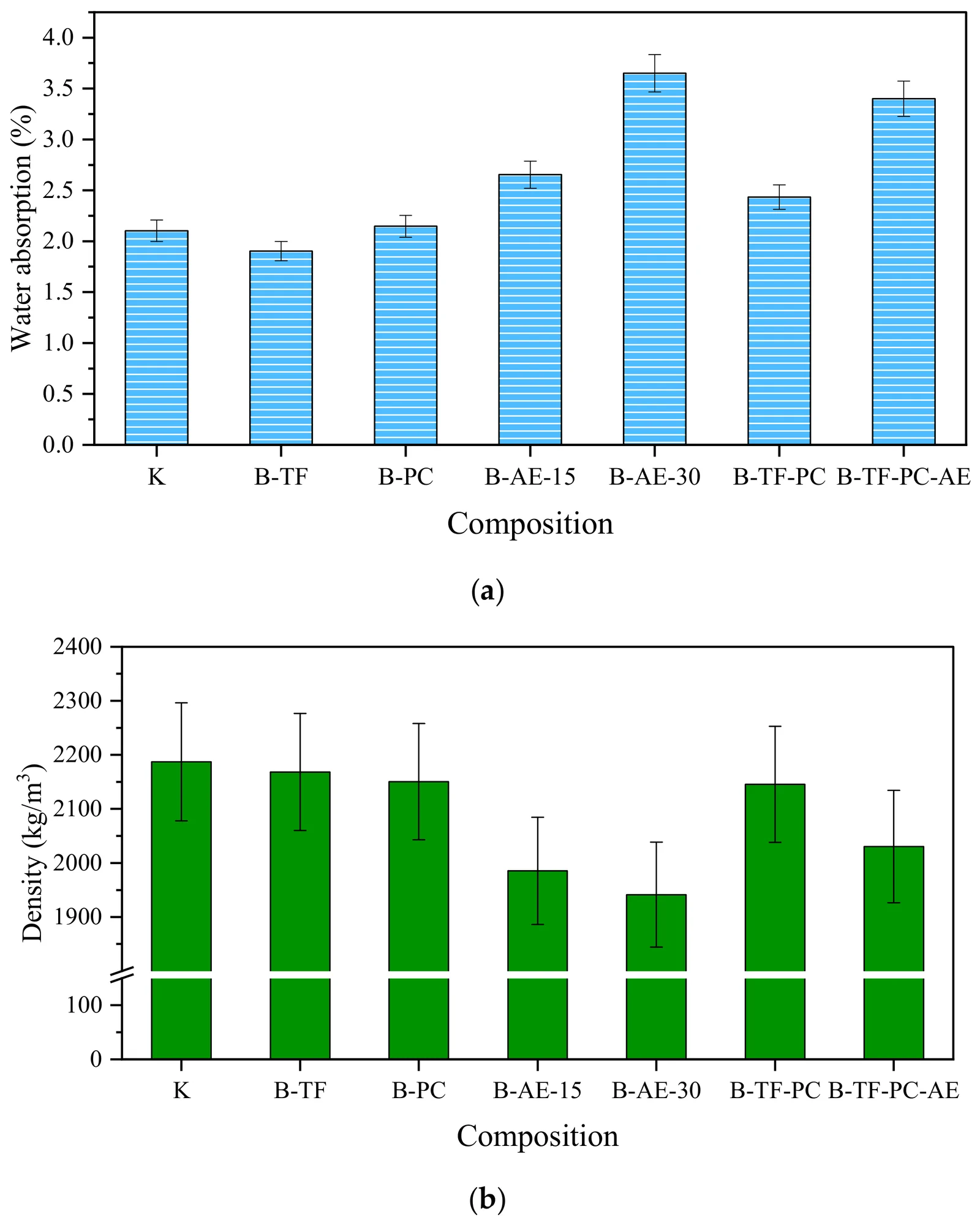
Water absorption (**a**) and density (**b**) of fine-grained concrete modified with mineral and chemical admixtures.

**Figure 23:**
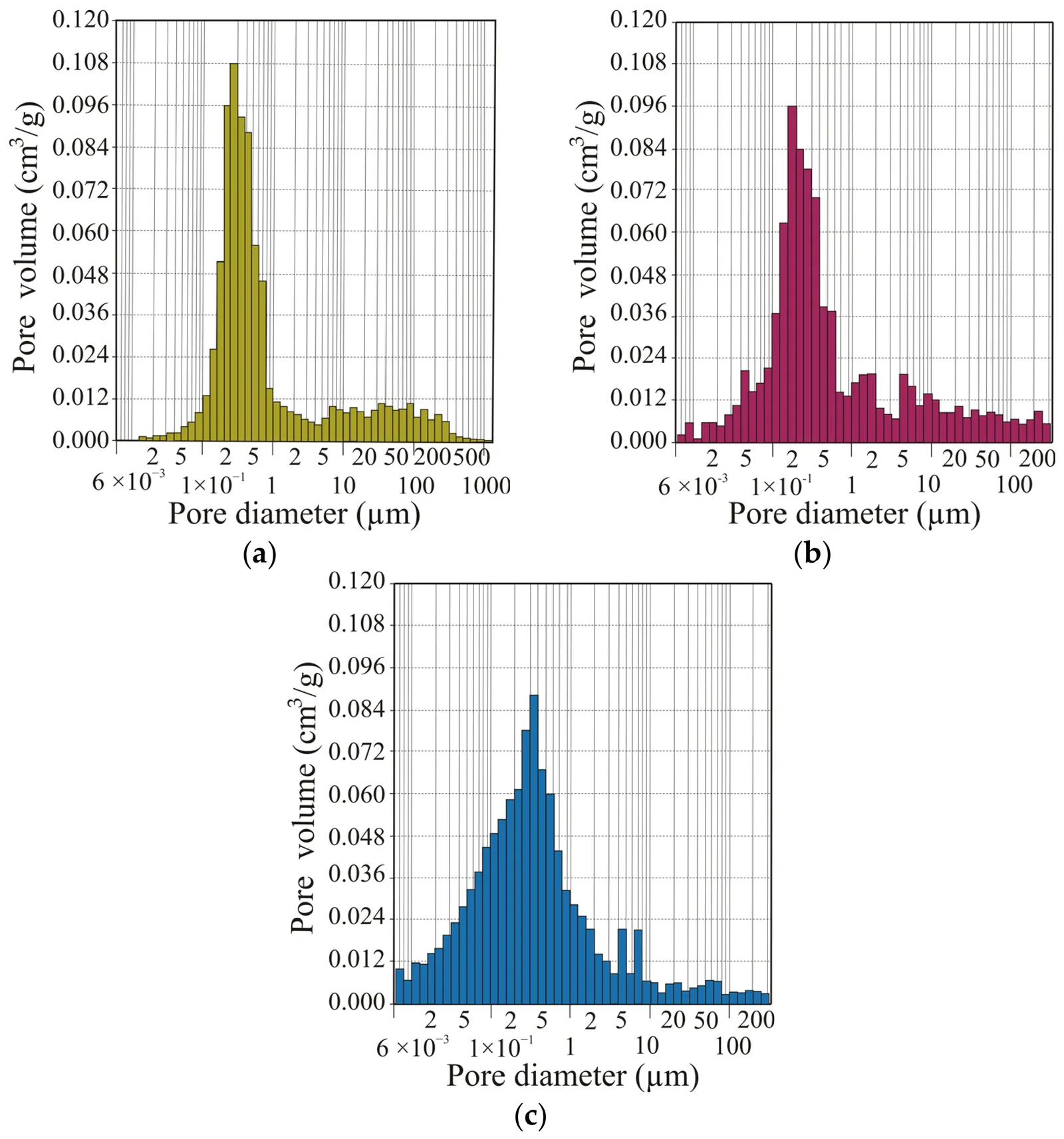
The pore size distribution (by volume) in the samples, depending on the used admixture: (**a**) B-TF, (**b**) B-TF-PC, (**c**) B-TF-PC-AE.

Analysis of the water absorption values showed (Figure 22) that the lowest water absorption over 24 h was estimated for B-TF (1.9%), K (2.1%), and B-PC (2.15%) samples. At the same time, the B-AE-15 and B-AE-30 compositions had the highest water absorption values of 2.65% and 3.65%, respectively. As shown, the highest density, 2187 kg/m^3^, was determined for control K samples. The use of TF slightly reduced the sample density to 2168 kg/m^3^. The combined use of TF and PC (composition B-TF-PC) increases water absorption to 2.43%. It decreases density to 2145 kg/m^3^, possibly because of the polycarboxylate ether-based superplasticizer’s ability to involve air during mixing [76]. In the case of the combined use of TF with the PC and AE admixtures (composition B-TF-PC-AE), the water absorption was 3.4%, which is higher than in composition B-TF-PC and in composition B-AE-15. The density of composition B-TF-PC-AE is 2030 kg/m^3^, which reflects the additional air voids introduced by the air-entraining admixture and is consistent with the pore-structure results (Figure 23).

### 3.4. Porosity

To assess the correspondence between the results of freeze–thaw resistance, porosity tests were performed, and based on these, the potential freeze–thaw resistance was calculated, allowing comparison of the results obtained in directly conducted tests with the calculated ones. Figure 23 shows how the admixtures affected the pore-size distribution (by volume) of the samples [77,78].

The mercury porosimeter measurements on the B-TF matrix sample showed that pores with diameters between 0.01 and 0.10 µm occupied 0.0099 cm^3^/g. Pores with diameters between 0.1 and 1 µm occupied the biggest portion of the volume (0.221 cm^3^/g), whereas pores with diameters between 1.0 and 10 µm and 10.0 and 100 µm occupied 0.071 and 0.029 cm^3^/g, respectively. Pores with diameters between 100 µm and 500 µm occupied 0.032 cm^3^/g.

Pores with a diameter between 0.001 and 0.01 µm (0.0014 cm^3^/g) and those with a diameter between 0.01 and 0.10 µm occupy 2 times higher volume (0.020 cm^3^/g) in the B-TF-PC matrix sample. As in the B-TF matrix sample, pores with a diameter between 0.1 and 1.0 µm occupy the largest volume, 0.248 cm^3^/g. Pores with diameters between 1.0 and 10 µm and 10.0 and 100 µm occupied larger volumes of 0.087 and 0.028 cm^3^/g, respectively, whereas the volume of these pores is a little higher than that of the B-TF matrix sample. Pores with diameters between 100 µm and 500 µm occupied 0.031 cm^3^/g. Generally, the pore volume in this sample is about 11% higher than in the B-TF matrix sample.

The B-TF-PC-AE matrix sample showed a two-fold increase in volume from 0.001 to 0.01 µm in diameter pores, reaching 0.0029 cm^3^/g. The pores’ volume, measured between 0.01 and 0.1 µm, increases by more than 1.5 times, reaching 0.03 cm^3^/g. The largest portion of the volume (0.31 cm^3^/g) is occupied by pores with diameters between 0.1 and 1 µm; the volume of pores between 1.0 and 10.0 µm increases and reaches 0.132 cm^3^/g. Pores with diameters between 10.0 and 100 µm occupied lower volumes of 0.03 cm^3^/g, respectively, and pores with diameters between 100 µm and 500 µm occupied 0.02 cm^3^/g.

In the sample containing both the PC and the AE, compared to the B-TF matrix sample, pores (0.001–0.01 µm) are observed, and both admixtures tend to increase by more than 2 times, while pores with a diameter ranging from 0.1 µm to 1 µm increase up to 28.7%. The volume of pores between 1.0 and 10.0 µm increased by 46.21% compared to B-TF matrix samples. When the B-TF-PC matrix and B-TF-PC-AE matrix are compared, the volume of pores between 1.0 and 10.0 µm increased by nearly 29.5%.

### 3.5. Potential Freeze–Thaw Resistance

Freeze–thaw resistance of most materials depends on pore geometry, specifically on the ratio of pores with diameters greater than 3 µm, which do not readily fill with water, to smaller pores, which absorb water and are susceptible to freezing under operating conditions [79,80]. The freeze–thaw resistance of construction materials is enhanced by pores with diameters greater than 3 µm. The total pore volume (PV) and the fraction of pores with diameters greater than 3 µm (P3) are used to calculate the potential freeze–thaw resistance number. The investigation [81,82] confirmed this clause. The authors conclude that pores with widths between 3 and 10 µm are common in construction materials that show no signs of deterioration in treated samples.

The total volume of pores (PV) and P3 are 0.3729 cm^3^/g and 19.03% for the B-TF sample, 0.4157 cm^3^/g and 22.61% for the B-TF-PC matrix sample, 0.5231 cm^3^/g and 30.73% for the B-TF-PC-AE matrix sample. According to the potential freeze–thaw resistance number calculation, the values are 54.25 for the B-TF matrix sample, 61.97 for the B-TF-PC matrix sample, and 72.83 for the B-TF-PC-AE matrix sample. Material samples with values more than 70 are considered durable, according to research in [62]. The Fc values of the B-TF and B-TF-PC matrix samples are below this limit, whereas the Fc value of the B-TF-PC-AE matrix sample exceeds it. The higher Fc value of the B-TF-PC-AE sample compared with the B-TF and B-TF-PC samples is consistent with its more favorable pore geometry, in agreement with the pore-structure results. Further research into the freeze–thaw resistance of concrete compositions confirms the calculations of the Maage factor and the prediction of freeze–thaw resistance.

As expected, the B-TF samples possess the lowest porosity and the lowest water absorption. In contrast, the B-TF-PC-AE samples exhibit the highest porosity and, correspondingly, the highest water absorption.

### 3.6. Compressive Strength Development of Fine-Grained Concrete

**Figure 24:**
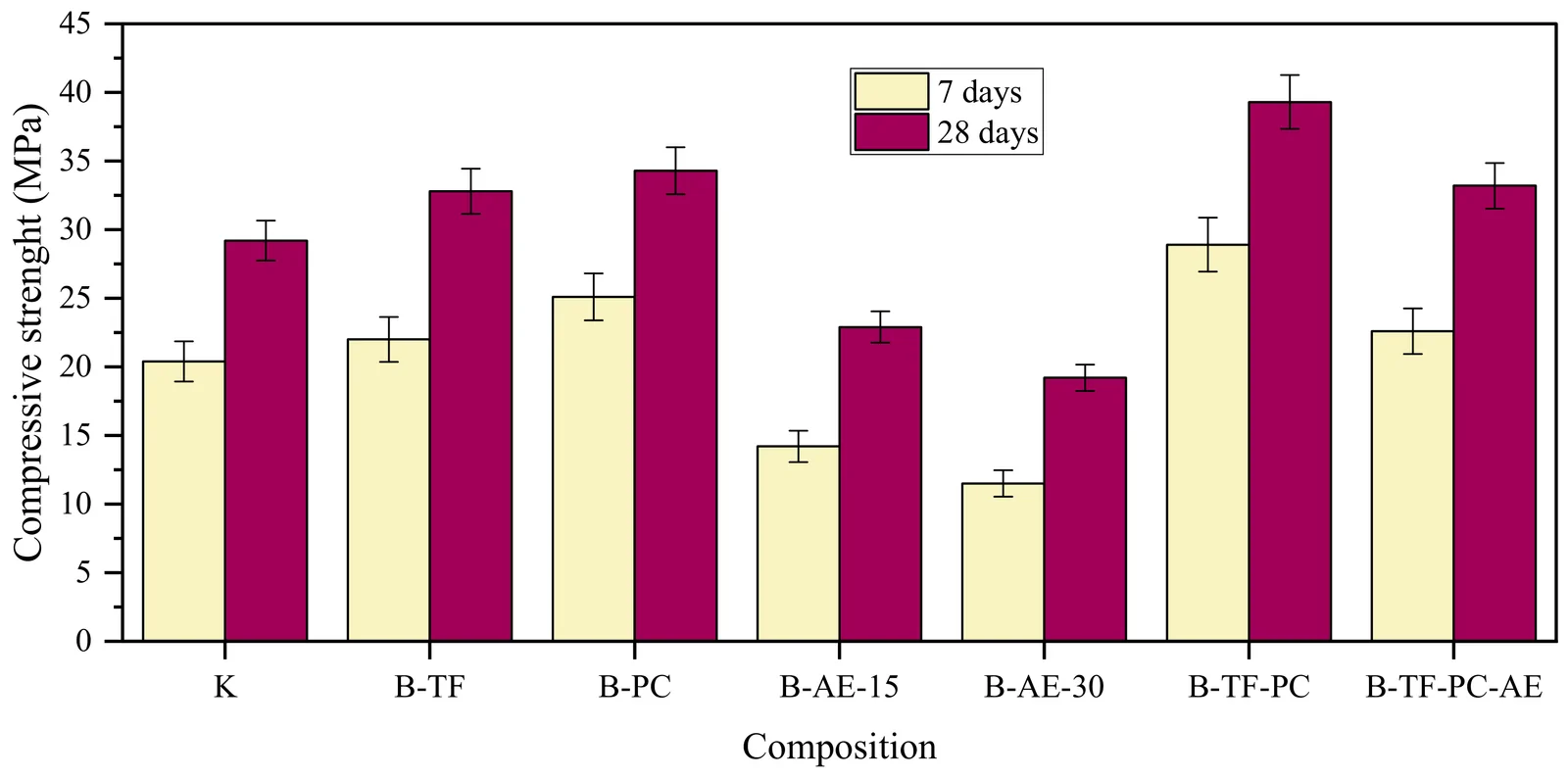
Compressive strength of fine-grained concrete mixtures after 7 and 28 days of curing.

Figure 24 illustrates the compressive strength development of the fine-grained concrete mixtures after 7 and 28 days. The compressive strength results demonstrated a clear dependence on the composition of the modifying paste and on the interactions among cement minerals, additives, and chemical admixtures. The reference mixture developed a compressive strength of 20.4 MPa at 7 days and 29.2 MPa at 28 days of curing. The incorporation of B-TF increased the strength to 22.0 and 32.8 MPa, respectively, indicating that the finely dispersed B-TF contributed to matrix refinement through both microfiller action and subsequent pozzolanic reactivity [32,33]. A more pronounced effect was observed for the composition B-PC, which achieved the highest strength among the single-modified systems, reaching 25.1 MPa at 7 days and 34.3 MPa at 28 days.

A similarly high performance was obtained for the closely related composition B-TF-PC, with corresponding strengths of 28.9 and 39.3 MPa. These results confirm that the combined use of TF and PC results in a denser, more homogeneous cementitious matrix, thereby enhancing strength development throughout the entire curing period [83]. By contrast, the introduction of the AE in the composition reduced compressive strength. At an AE dosage of 0.15% in the B-AE-15 composition, the strength decreased to 14.2 MPa after 7 days and 22.9 MPa after 28 days, while a further increase in B-AE-30 reduced the strength values to 11.5 and 19.2 MPa, respectively. This behavior is consistent with the formation of an additional air-void system, which is essential for improving freeze–thaw resistance but inevitably reduces the effective load-bearing cross-section of the hardened concrete.

Nevertheless, the ternary system B-TF-PC-AE maintained a relatively high compressive strength of 22.6 MPa at 7 days and 33.2 MPa at 28 days, exceeding the control K mixture despite the presence of entrained air. This finding indicates that the synergistic action of TF and PC can effectively offset the negative effect of AE on strength. Air entrainment inevitably lowers strength by adding porosity to the matrix, the reduction reported for air-entrained concrete being of the order of 5–17% and increasing with the air content [78,84]. In the present system, this loss is compensated rather than avoided. The superplasticizer lowers the effective water-to-cement ratio and compacts the interfacial transition zone [77], and increasing its content in air-entrained concrete has been shown to progressively offset the strength penalty of air entrainment [84]. In parallel, the fine mechanically activated ash fills the capillary pores and refines the matrix through its filler and early pozzolanic action, minimizing void spaces and densifying the paste [32,85]. The strength reserve created by the denser TF–PC matrix therefore outweighs the loss caused by the entrained air, so that B-TF-PC-AE, despite its higher air content, still exceeds the plain reference K. Overall, the PC mixtures, particularly B-TF-PC and B-TF-PC-AE, exhibited the most favorable performance, combining high compressive strength with the expected durability benefits associated with a properly developed air-void structure.

### 3.7. Freeze–Thaw Resistance

**Figure 25:**
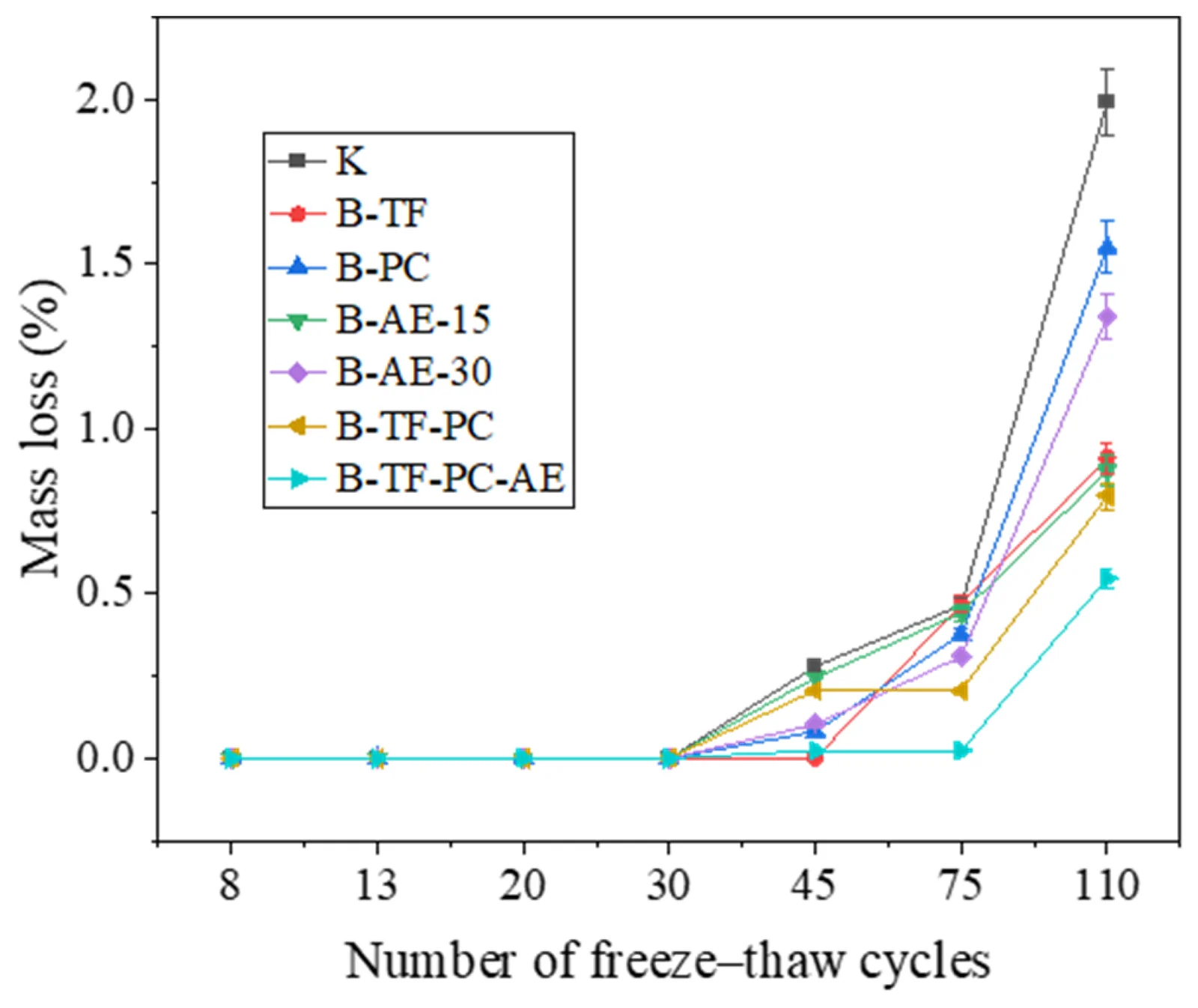
Mass loss of fine-grained concrete during accelerated freeze–thaw cycling in a 5% NaCl solution. Error bars represent ± one standard deviation.

**Figure 26:**
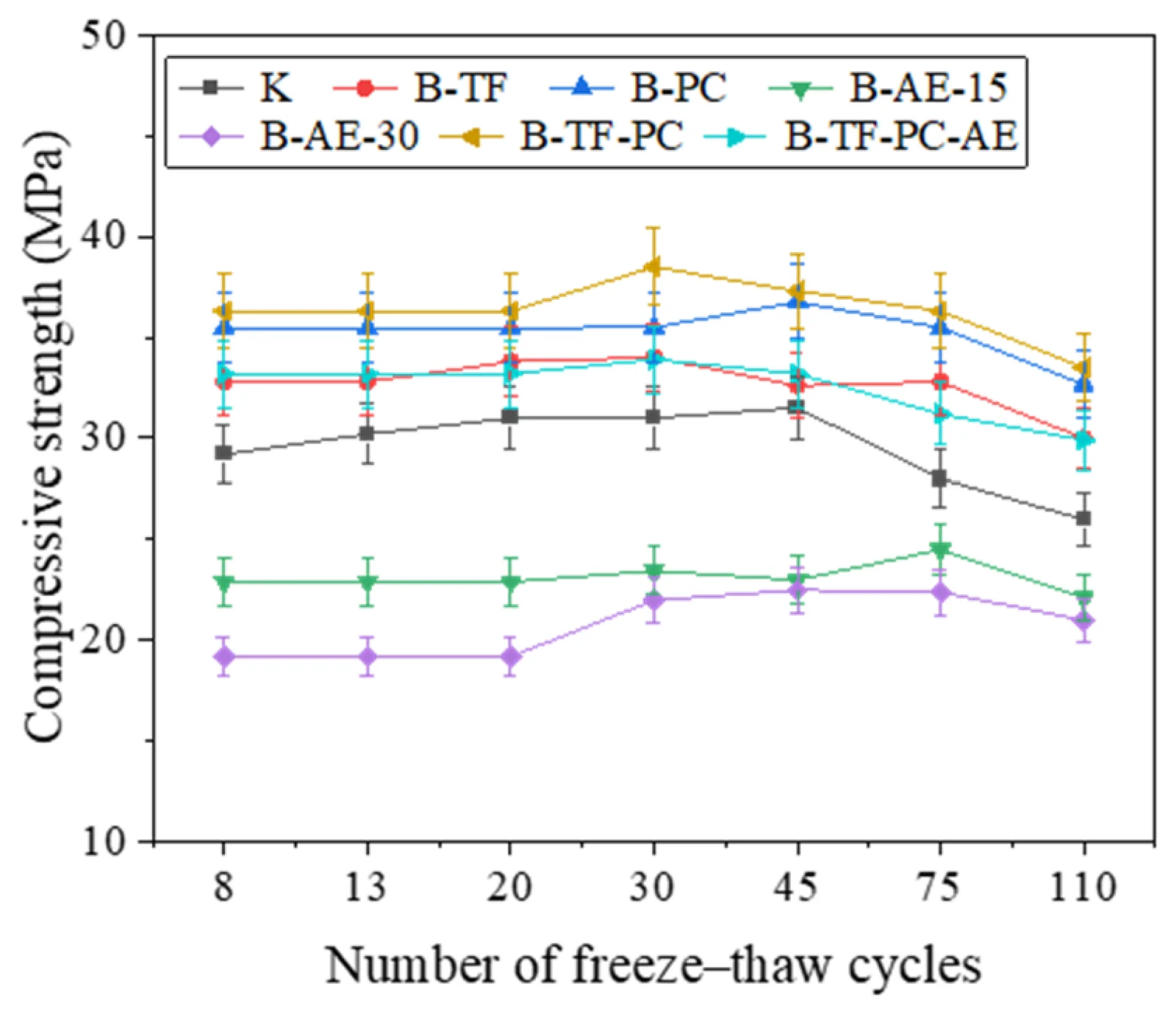
Changes in the compressive strength of fine-grained concrete as a function of the number of accelerated freeze–thaw cycles in a 5% NaCl solution. Error bars represent ± one standard deviation.

The freeze–thaw resistance test results presented in Figure 25 and Figure 26 demonstrate that all tested fine-grained concrete compositions retained relatively high resistance to accelerated cyclic freezing and thawing in a 5% NaCl solution. Up to 30 accelerated freeze–thaw cycles, no measurable mass loss was recorded for any of the compositions. All tested compositions remained stable at these stages, with no visible signs of surface degradation (Figure 25). After 45 accelerated freeze–thaw cycles, all compositions exhibited only minor mass losses not exceeding approximately 0.30%, indicating limited surface degradation at this stage. After 75 accelerated freeze–thaw cycles, mass loss increased moderately, remaining within approximately 0.20–0.47% for most compositions. The highest mass losses at this stage were recorded for the reference composition K and the compositions B-PC, B-AE-15, and B-AE-30.

In contrast, the B-TF and B-TF-PC-AE compositions demonstrated the lowest mass losses. This behavior can be attributed to the denser and more uniform pore structure of the TF-containing and combined admixture compositions, as confirmed by water absorption and porosity tests. The denser matrix limits water ingress and reduces the volume of freezable water within the capillary pore system, thereby improving resistance to repeated freeze–thaw cycles, as frost resistance is closely related to pore structure, capillary connectivity, water absorption, and mass loss under freeze–thaw exposure [86].

At the final stage, after 110 accelerated freeze–thaw cycles, the compositional differences became more pronounced. The highest mass loss was recorded for the control composition K, amounting to approximately 1.99%. The B-PC composition also showed a comparatively high mass loss of approximately 1.56%. For the B-AE-30 composition, the value was approximately 1.34%, while the lowest mass losses were observed in the mixes B-TF-PC-AE (0.55%), B-TF-PC (0.8%), and B-AE-15 (0.87%). A slightly higher mass loss (0.91%) was observed for B-TF. However, it can be noted that although the mass losses after the cycles were similar for the B-AE-15 and B-TF compositions, the mechanical properties of the B-TF composition differ markedly—32.8 MPa for B-TF and 22.9 MPa for B-AE-15 compositions.

The interpretation of the results was based on the combined assessment of mass loss, compressive strength retention, and visual surface condition. After 110 accelerated freeze–thaw cycles, the mass loss of all compositions remained within the accepted limit of 2%. At the same time, no critical visual damage, such as cracking, spalling, or severe surface scaling, was observed.

The change in compressive strength depending on the number of freeze–thaw cycles is shown in Figure 26. The results showed that, in some cases, a slight increase rather than a decrease in strength was observed at intermediate stages of testing. That was most pronounced for the B-PC, B-AE-30, and B-TF-PC compositions, for which strength increased by 30–45 cycles. For the B-TF-PC composition, the maximum strength was approximately 38 MPa, the highest among all the mixtures tested. The B-PC composition also maintained a high strength of approximately 35–37 MPa.

The control composition K and the B-TF compositions were characterized by relatively stable strength at the initial and intermediate stages of testing, after which a decrease was observed by 110 cycles.

After 110 cycles, the strength values for K and B-TF were approximately 26 and 30 MPa, respectively. For the air-entrained compositions, the absolute strength was lower than that of the control K and B-PC-containing compositions, particularly noticeable for the B-AE-15 composition, where values ranged from approximately 22.1 MPa. The lowest strength over the entire test cycle was observed for the B-AE-30 mixture, although it also showed a temporary increase in strength by 75 cycles. This temporary strength increase may be associated with continued hydration during cyclic exposure, since slow cement hydration may continue even when concrete is subjected to freeze–thaw cycling; however, additional microstructural evidence would be required to confirm this mechanism [87].

The combined use of admixtures and the presence of B-TF in the composition B-TF-PC-AE demonstrated consistent performance throughout the test: strength was initially around 33 MPa and remained at approximately 31 MPa after 110 cycles. That demonstrates a fairly effective combination of mineral and chemical modification during prolonged frost exposure.

The changes in compressive strength shown in Figure 26 indicate that a temporary strength increase was observed after approximately 30–40 cycles for most compositions, except B-PC. This effect may be related to continued hydration during cyclic exposure and possible partial filling of microdefects by newly formed hydration products. However, this mechanism should be considered hypothetical without additional microstructural evidence. After 60–70 cycles, the beneficial effect became less pronounced, indicating that continued cyclic exposure progressively reduced the structure’s ability to compensate for damage accumulation. The temporary strength gain is consistent with continued hydration during cyclic exposure, in which slowly reacting fly ash and clinker progressively fill microdefects with new hydration products; the parallel low water absorption and refined pore structure of these compositions support this interpretation.

The presence of only one PC in the composition markedly worsens the samples’ resistance to freezing and thawing cycles [88]. The combination of pores created by both additives—PC and AE does not allow the strength of the samples to decrease substantially. It is possible that ground ash, due to the irregular shape of its particles, promotes a uniform distribution of air bubbles in the sample. That is also confirmed by the potential freeze–thaw resistance number calculations, which show that samples with fly ash and superplasticizer have a lower factor than those with both additives when used together.

Overall, the strength results confirm that all tested compositions remained functional throughout the accelerated freeze–thaw exposure and retained satisfactory mechanical performance after 110 cycles in a 5% NaCl solution.

The action of the 5% NaCl solution also has a chemical side. Part of the chloride penetrating into the concrete is bound by the aluminate phases in the form of Friedel’s salt (Equation (5)):

C_3_A + CaCl_2_ + 10H_2_O → C_3_A·CaCl_2_·10H_2_O (5)

while the remaining free chloride interacts with portlandite (Equation (6)),

Ca(OH)_2_ + 2NaCl → CaCl_2_ + 2NaOH (6)

and promotes its gradual leaching from the surface layers. The loss of Ca(OH)_2_ increases the capillary porosity of the surface zone, and together with the higher osmotic pressure and degree of saturation this explains why surface scaling develops although the total mass loss stays below 2%. In the compositions with TF, part of the portlandite is consumed by the pozzolanic reaction and converted into insoluble C–S–H, and the pore structure becomes finer, so the ingress of the salt solution is reduced. That agrees with the better strength retention of the ternary composition and its lowest mass loss (0.55%).

Within the 110 accelerated cycles applied here, the air-entrained compositions showed no clear advantage over the B-TF and B-TF-PC mixtures, as the mass loss of all mixtures remained below 2%. It should also be noted that the adopted regime employs a 5% NaCl solution, which is considerably more severe than freezing and thawing in water: under comparable numbers of cycles, salt–frost exposure has been reported to increase the losses in strength and mass by a factor of 1.5–3 and 3–5, respectively, in salt solutions relative to fresh water [89], and the aggravating effect of salt solutions on freeze–thaw damage has been confirmed for pavement concretes [86]. Accordingly, standardized salt–frost procedures employ short exposures: the RILEM CDF reference method is assessed after 28 cycles with sodium chloride solution [90], while in CEN/TS 12390-9 [63] the reference duration of the de-icing-salt test is 56 cycles, an exposure commonly adopted in studies of superplasticized and air-entrained concretes [88]; the 110 salt–frost cycles applied here thus correspond to twice the standard European exposure and four times the RILEM reference duration. The protective role of the entrained air-void system typically becomes decisive under more prolonged exposure [20], so verification of B-AE-15, B-AE-30 and B-TF-PC-AE at higher cycle counts, such as 200–300 cycles, is identified as a direction for future research.

## 4. Conclusions

1. Both mineral and chemical admixtures markedly influenced the fresh-state behavior, setting characteristics, and early hydration of the investigated cement-based systems, altering the kinetics of hydration and structure formation. Mechanically activated fly ash (TF) exhibited a more pronounced modifying effect than untreated fly ash (UF): pastes with TF and both chemical admixtures showed up to 5% lower spread and up to 11% lower initial setting time than the corresponding UF pastes, and a smaller decrease in the maximum exothermic temperature, confirming that mechanical activation partially compensated for the retarding effect of the combined admixtures and promoted a more intense early hydration.
2. The microstructural and phase analyses supported the macroscopic results. Untreated fly ash largely preserved its original spherical morphology and showed limited participation in structure formation, whereas mechanically activated fly ash produced a finer, more uniformly distributed structure with a higher degree of interaction with the hydrated cement matrix. The superplasticizer primarily densified the matrix, while the air-entraining admixture generated larger reserve pores beneficial for freeze–thaw resistance.
3. Fine-grained concrete containing mechanically activated fly ash reached a 28-day compressive strength of 32.8 MPa, increasing to 39.3 MPa with the superplasticizer; the simultaneous use of the superplasticizer and the air-entraining admixture gave 33.2 MPa, consistent with the higher total pore volume, showing that the synergy of TF and PC effectively offsets the strength loss caused by air entrainment. The lowest water absorption (1.9%) was obtained for the TF composition, rising to 2.43% with the superplasticizer and to 3.4% for B-TF-PC-AE. The combined superplasticizer and air-entraining admixture increased the volume of 0.01–0.10 µm pores threefold and of 1.0–10 µm pores by up to 46.21%; the corresponding potential freeze–thaw resistance number (Fc) rose from 54.25 (B-TF) to 61.97 (B-TF-PC) and 72.83 (B-TF-PC-AE), confirming that a higher fraction of 3–10 µm pores improves freeze–thaw resistance.
4. All investigated concretes exhibited relatively high durability under accelerated cyclic freezing and thawing in a 5% NaCl solution: after 110 cycles the mass loss remained limited, with the lowest value of 0.55% for the B-TF-PC-AE composition, and no critical loss of compressive strength or unacceptable visual damage was observed. Overall, the combined use of mechanically activated fly ash, superplasticizer, and air-entraining admixture proved an effective approach for tailoring the structure and performance of fine-grained concrete, with the B-TF-PC and B-TF-PC-AE compositions showing the most balanced combination of fresh-state properties, strength, refined pore structure, and freeze–thaw durability.

## Figures and Tables

**Table 1:** Chemical composition of untreated (UF) and mechanically activated (TF) fly ash (wt.%).

SiO_2_	Al_2_O_3_	Fe_2_O_3_	CaO	MgO	Na_2_O	K_2_O	P_2_O_5_	TiO_2_	MnO	SO_3_	LOI
57.03	31.71	1.66	1.12	0.45	0.33	1.10	0.10	1.16	0.02	0.12	5.2
58.71	32.57	1.66	1.39	0.35	0.30	0.98	0.05	1.10	0.01	0.10	4.2

Notes: the first data row corresponds to untreated fly ash (UF); the second data row corresponds to mechanically activated fly ash (TF).

**Table 2:** Physical and mechanical properties of fine aggregate.

Property	Value
Fineness modulus	2.66
Density (kg/m^3^)	2620
Bulk density (kg/m^3^)	1630
Void ratio (%)	38.2
Clay and dust content (%)	0.9
Water demand (%)	5.4

**Table 3:** Mix design of cement paste systems used for exothermic analysis.

Mix ID	Cement *, %	UF, %	TF, %	PC *, %	AE, %	W/PC
C-0	100	-	-	-	-	0.27
UF-5	95	5	-	-	-	0.27
UF-10	90	10	-	-	-	0.27
UF-15	85	15	-	-	-	0.27
TF-5	95	-	5	-	-	0.27
TF-10	90	-	10	-	-	0.27
TF-15	85	-	15	-	-	0.27
PC-0.5	100	-	-	0.5	-	0.27
PC-1	100	-	-	1	-	0.27
PC-1.5	100	-	-	1.5	-	0.27
AE-15	100	-	-	-	0.15	0.27
AE-30	100	-	-	-	0.3	0.27
AE-45	100	-	-	-	0.45	0.27
PC-AE-15	100	-	-	1	0.15	0.27
PC-AE-30	100	-	-	1	0.3	0.27
PC-AE-45	100	-	-	1	0.45	0.27
UF-PC-AE	95	5	-	1	0.15	0.27
TF-PC-AE	95	-	5	1	0.15	0.27

Notes: C-0—reference (control) cement paste; UF—untreated fly ash; TF—mechanically activated fly ash; PC—polycarboxylate superplasticizer; AE—air-entraining admixture; the number after the component denotes its content or dosage in % by mass of cement (e.g., UF-5 = 5% untreated fly ash, PC-0.5 = 0.5% superplasticizer, AE-15 = 0.15% air-entraining admixture). W/PC—water-to-cement ratio. * Cement content is expressed as wt.% of the total binder (cement + fly ash); PC dosage is expressed as wt.% of cement.

**Table 4:** Mix proportions of fine-grained concrete mixtures used in the experimental study.

Mix ID	Cement, kg/m^3^	Sand, kg/m^3^	TF, kg/m^3^	PC, kg/m^3^	AE, kg/m^3^	Water, kg/m^3^
K	450	1550	-	-	-	230
B-TF	428	1550	23	-	-	230
B-PC	450	1550	-	4.5	-	230
B-AE-15	450	1550	-	-	0.675	230
B-AE-30	450	1550	-	-	1.35	230
B-TF-PC	428	1550	23	4.5	-	230
B-TF-PC-AE	428	1550	23	4.5	0.675	230

Notes: K—reference (control) concrete without admixtures; B—base fine-grained concrete; TF—mechanically activated fly ash; PC—polycarboxylate superplasticizer; AE—air-entraining admixture; the trailing number denotes the air-entraining admixture dosage (15 and 30 correspond to 0.15% and 0.30% by mass of cement, respectively).

## Data Availability

The original contributions presented in the study are included in the article, further inquiries can be directed to the corresponding authors.
